# Pecan kinome: classification and expression analysis of all protein kinases in *Carya illinoinensis*

**DOI:** 10.48130/FR-2021-0014

**Published:** 2021-08-18

**Authors:** Kaikai Zhu, Pinghua Fan, Hui Liu, Juan Zhao, Pengpeng Tan, Zhenghai Mo, Fangren Peng

**Affiliations:** 1 Co-Innovation Center for Sustainable Forestry in Southern China, Nanjing Forestry University, Nanjing, Jiangsu 210037, China; 2 State Key Laboratory of Crop Genetics and Germplasm Enhancement, Ministry of Agriculture and Rural Affairs Key Laboratory of Biology and Germplasm Enhancement of Horticultural Crops in East China, College of Horticulture, Nanjing Agricultural University, Nanjing, Jiangsu 210095, China; 3 Institute of Botany, Jiangsu Province and Chinese Academy of Sciences, Nanjing 210014, China

**Keywords:** *Carya illinoinensis*, Classification, Co-expression networks, Expression patterns, Kinome

## Abstract

Protein kinases (PKs) are involved in plant growth and stress responses, and constitute one of the largest superfamilies due to numerous gene duplications. However, limited PKs have been functionally described in pecan, an economically important nut tree. Here, the comprehensive identification, annotation and classification of the entire pecan kinome are reported. A total of 967 PK genes were identified from the pecan genome, and further classified into 20 different groups and 121 subfamilies using the kinase domain sequences, which were verified by phylogenetic analysis. The receptor-like kinase (RLK) group contained 565 members, which constituted the largest group. Gene duplication contributed to the expansion of pecan kinome, 169 segmental duplication events including 285 PK genes were found, and the *Ka*/*Ks* ratio revealed they experienced strong negative selection. The RNA-Seq data of PK genes in pecan were further analyzed at the subfamily level, and different PK subfamilies performed various expression patterns across pecan embryo development or drought treatment, suggesting PK genes in pecan are involved in embryo development and drought stress response. Taken together, this study provides insight into the classification, expansion, evolution, and expression of pecan PKs. Our findings regarding expansion, expression and co-expression analyses lay a good foundation for future research to understand the roles of pecan PKs, and more efficiently determine the key candidate genes.

## INTRODUCTION

Reversible phosphorylation is a common type of post-translational modification, which is catalyzed by protein kinases (PKs), widely existing in living organisms^[1]^. PKs regulate the activity of downstream target proteins via transferring the phosphates to phosphorylate specific amino acids including serine, threonine or tyrosine as molecular switches^[2]^. PKs constitute a super gene family with a large number of members in plants, and the entire PKs in a genome are defined as the kinome. More than 1000 *PK* genes were found in *Arabidopsis*, representing about 4% of the genome^[3]^. However, only 518 putative PKs were identified in the human genome, which constitutes 1.7% of entire human genes^[4]^.

In general, PKs have a catalytic domain ranging from 250 to 300 amino acid residues. This superfamily was first classified into various subfamilies based on the phylogenetic analysis of the catalytic domain sequences^[5]^. In recent years, hundreds of plant genome sequences have been released, providing an excellent opportunity in the understanding of the evolutionary history of plant PKs. Kinomes from 25 plant species were identified and further classified into nine major groups with 115 families, and the PKs experienced huge expansion in flowering plants^[6]^. In soybean, 2,166 putative PKs were found, and divided into 19 groups and 122 subfamilies^[7]^. In the grapevine kinome, 1,168 PK genes were classified into 20 main groups and 121 subfamilies, the RLK-Pelle was the largest group with 872 PKs^[8]^. The huge expansion of kinome in flowering plants is due to gene duplication and a good retention rate of duplicates in some groups, especially the RLK-Pelle group^[9]^. Only four Interleukin Receptor-Associated Kinase (IRAK) genes have been found in the human genome, which perform a close relationship with plant RLK-Pelle group^[10]^.

Functional characterization studies of plant PK genes have mainly occurred in model plants such as *Arabidopsis* and rice, and PKs have been proven to play key roles involved in various biological processes^[3, 11, 12]^. However, few PK genes have been functionally analyzed in non-model plants, especially in perennial woody plants.

The pecan tree [*Carya illinoinensis* (Wangenh.) K. Koch] is a well known commercially cultivated nut tree worldwide, which is native to North America and Mexico^[13]^. Pecan is a member of the Juglandaceae family in the genus *Carya*, and the delicious nuts are a good source of unsaturated fatty acids, flavonoids and protein for human benefit seeing an increase in consumption in recent years^[14]^. In 2018, the United States of America, produced over 130,000 tons of pecan nuts, with a total production value approaching $600 million (https://www.nass.usda.gov). Recently, the release of the pecan genome and transcriptome data has allowed characterization of the pecan kinome, duplication events, and their expression patterns under different conditions^[15]^. In the current study, 967 pecan PKs were identified and further classified into different groups and subfamilies. Conserved domain sequence features and phylogenetic relationships of different subfamily members were also evaluated. Subsequently, the expression patterns and co-expression networks of various subfamilies were analyzed to more efficiently determine the key members. Collectively, the comprehensive annotation of pecan PK genes and expression files helps us to understand the potential roles of pecan protein kinases.

## RESULTS

### Genome-wide identification of protein kinases in pecan

**Figure 1:**
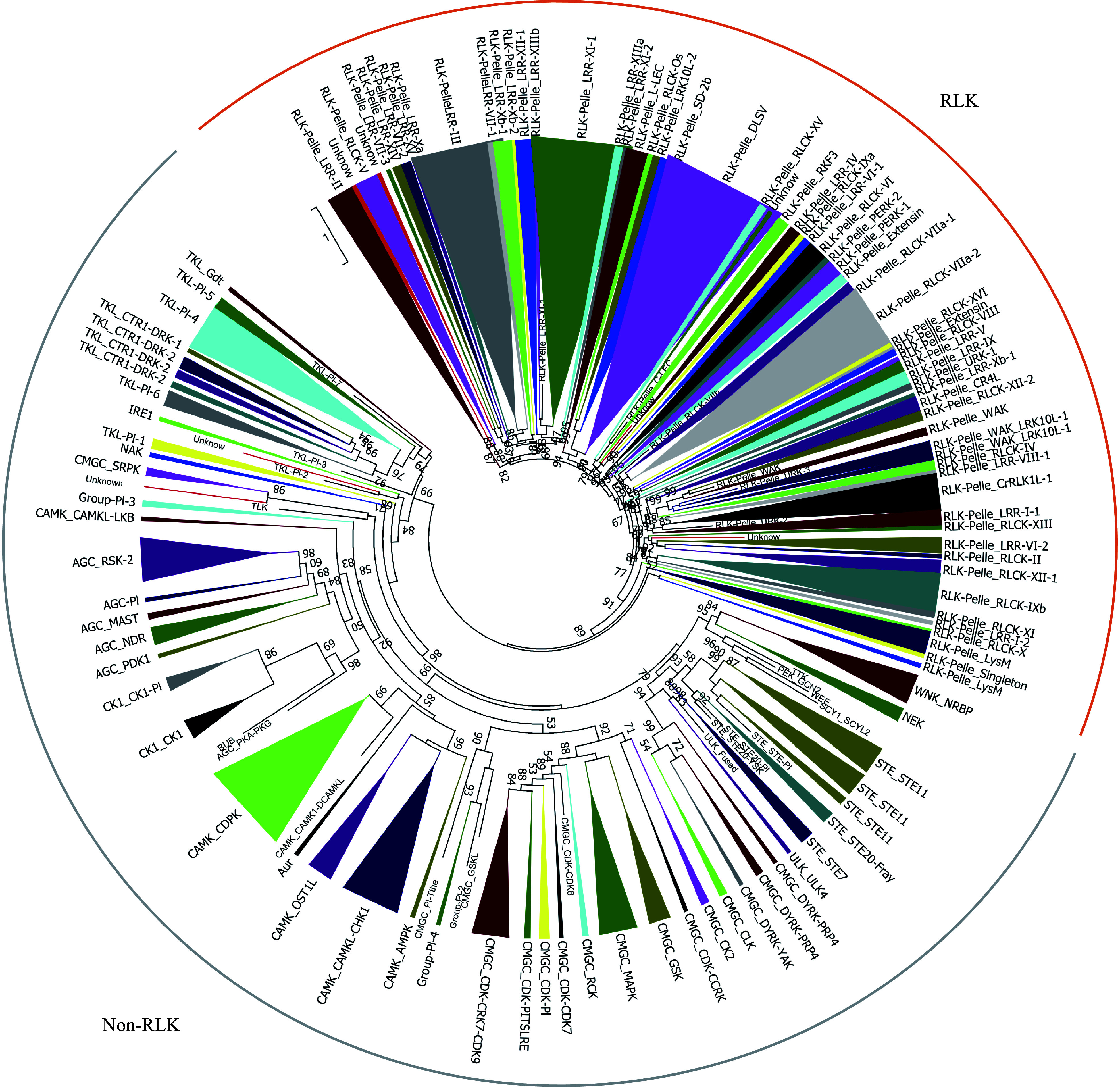
Phylogenetic analyses and classification of PKs identified in the pecan genome. The phylogenetic tree of the 967 PKs in pecan was constructed by kinase domain sequences and classified into 121 subfamilies. Branches were colored to represent two major clades, the RLK clade was marked as orange, and the non-RLK clade was marked as grey.

All pecan protein sequences were aligned against the kinase domains by HMMER, and a total of 1,112 candidates were identified following exclusion of redundant sequences. The coverage of kinase domains of 1,112 protein alignments were then evaluated, and 967 were identified as typical PKs which contained at least 50% of the domain alignments (Supplemental Table S1)^[6]^. These pecan PKs were classified into different groups and families using HMM search method, and 11 were found to provide a different result through phylogenetic analysis (Supplemental Fig. S1). The 11 PK genes also had low E-values, were not clustered with any of the known PK subfamilies, and placed in an unclassified cluster (named as 'UNKNOWN'). The remaining 956 pecan PKs were further classified into 121 subfamilies, corresponding to 20 groups (Supplemental Fig. S1). The receptor-like kinase (RLK) group contained 59 subfamilies and 565 members, which accounted for 58.4% and comprised the largest group in pecan kinome. The other six major groups included CAMK (94), CMGC (87), TKL (65), STE (46), AGC (39), CK1 (17). Similar to the groups, the size of the subfamilies was also greatly variable and varied from one to 64 genes (Fig. 1).

To gain insight into the evolutionary relationships of the PK families, a phylogenetic tree was built using the kinase domain sequences from four plant species including *Arabidopsis*, pecan, grape, and pineapple genomes (Supplemental Fig. S2). The pecan was phylogenetically closer to grape and *Arabidopsis*, the three species belonged to the dicotyledons, and the pineapple was a monocotyledon. Twenty-three PK subfamilies in pecan contained one member, examples being: CMGC_CDK-CDK8, CMGC_PI-Tthe, PEK_GCN2, RLK-Pelle_C-LEC, RLK-Pelle_RLCK-VIIb. Interestingly, these subfamilies were also highly conserved in grape, *Arabidopsis* and pineapple kinomes, suggesting the expansion of these subfamilies was limited. TKL-Pl-8 was only found in pineapple and grape, and absent in pecan and *Arabidopsis*, while the SCY1_SCYL1 subfamily was absent in pecan and pineapple kinomes. The RLK-Pelle_DLSV was the largest subfamily in all four kinomes, 158 subfamily members were identified in grape, while only 64 and 41 were found in pecan and pineapple kinomes, respectively. The RLK-Pelle_LRR-XI-1 was comprised of 52 members in pecan, whereas *Arabidopsis* and pineapple contained 33 and 27 members respectively.

### Characterization of pecan PK properties

The identified PK proteins consisted of 149−1,634 amino acids, and the predicted molecular weight (MW) values varied from 17.24 kDa (CIL0895S0070) to 180.1 kDa (CIL1226S0042). The theoretical isoelectric points (pI) of the PK proteins ranged from 4.49 to 9.85, indicating they might function in various microenvironments.

To analyze the structural diversity of the pecan PKs in various subfamilies, the intron numbers of PK genes were collected. The intron numbers of genes in the pecan kinome varied widely from 0 to 30, with 127 being intronless, while 205 (21.2%) of them possessed at least ten introns. The average intron number of the 967 pecan kinase genes was six, and *CIL1158S0028* contained the largest number of introns (Supplemental Table S2). After comparing the exon/intron arrangement in various subfamilies, we found that intron numbers in nine subfamilies were highly conserved. For example, all nine genes in RLK-Pelle_CR4L subfamily were intronless, and all RLK-Pelle_LRR-XIIIb members contained 26 introns. However, the gene structure of PK genes in some subfamilies were highly variable; for example, 13 CAMK_CAMKL-CHK1 subfamily members contained more than nine introns, whereas the intron numbers of the 17 remaining members were less than two.

**Figure 2:**
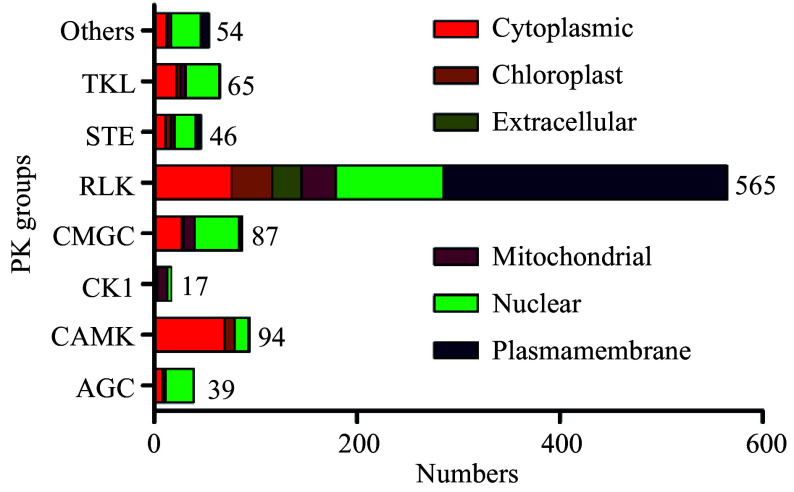
Subcellular localizations of pecan PK genes in different groups predicted by CELLO.

The subcellular location information can also be used to predict gene functions, and the subcellular localizations of pecan PK proteins were predicted according to CELLO software (Supplemental Table S2). Based on the results obtained, we found 30.8% (298/967) of PKs in pecan were predicted to localize to the plasma membrane, and most of them (280) were members of RLK groups. Intriguingly, 71.8% of PKs in the AGC group were localized to the nucleus, while 73.4% of members in the CAMK group were localized to the cytoplasm. Over half of PK genes in the CK1 group were localized to the mitochondria, and about 50% of CMGC and TKL members were predicted to localize to the nucleus (Fig. 2). Members in only 18 subfamilies were predicted to have the same subcellular locations, however, other PK genes within the same subfamilies were localized to different cellular compartments (Supplemental Table S2).

Conserved domains of 967 PK proteins in pecan were detected, and about half of them (489/967) only contained one kinase catalyst (Supplemental Table S3). The remaining 487 PKs with additional domains were investigated, and found in AGC (82.05%), TKL (72.31%), CAMK (63.83%), RLK (54.34%), STE (26.09%), and CMGC (10.34%) groups, indicating that different groups performed multiple domain compositions. Members in each subfamily commonly showed similar domain organizations, for example, all RLK-Pelle_L-LEC members contained an additional Lectin_legB domain, suggesting that they might share a common evolutionary history. In total, 43 PKs were identified from 16 subfamilies, which had two conserved kinase domains, including 20 AGC, 15 RLK and six CMGC group members (Supplemental Table S4).

### Functional prediction of pecan PKs

**Figure 3:**
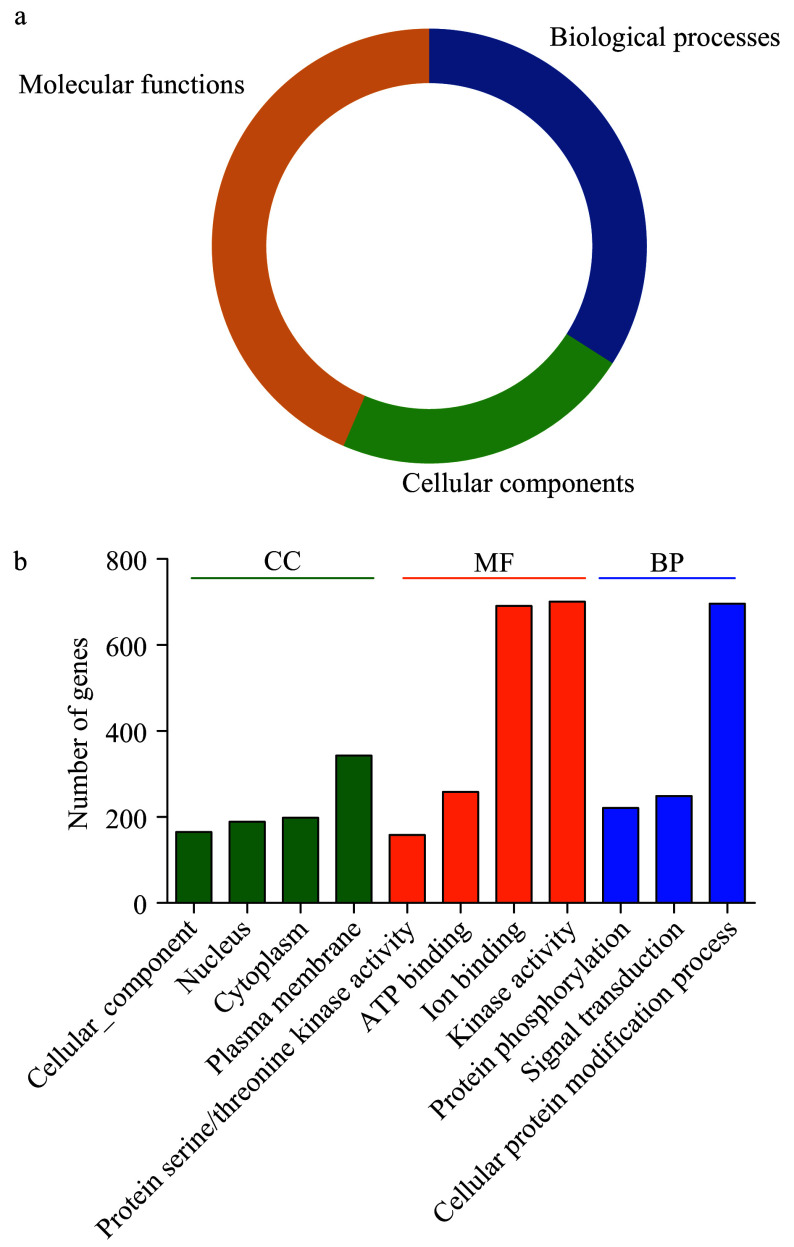
Gene ontology (GO) analyses of pecan PKs. (a) Pie charts indicated the relative proportion of the GO terms in the pecan kinome. (b) Detailed annotations in the different biological process (BP), cellular component (CC), and molecular function (MF) categories.

Three main gene ontology (GO) categories include biological processes (BP), molecular functions (MF) and cellular components (CC), GO analysis can help to predict the various functions of PK proteins. Therefore, the GO annotations of 967 PKs in the pecan kinome were investigated (Fig. 3 and Supplemental Table S5), it was found that these PKs were involved in the three GO categories. The largest fractions of the GO terms (43.56%) were related to molecular functions, and 34.05% were associated with biological processes, while only 22.4% were involved in cellular components (Fig. 3a).

Functional GO terms for the 967 pecan PKs were assessed, and the top eleven GO terms identified in more than 100 PKs were listed (Fig. 3b). According to the BP results, 71.98% PKs (696) in pecan were associated with cellular protein modification process (GO:0006464), 221 and 249 were related to protein phosphorylation (GO:0006468) and signal transduction (GO:0007165), respectively, suggesting most PKs participated in various biological processes by modifying protein functions. The top four GO terms in the MF category included kinase activity (GO:0016301), ion binding (GO:0043167), ATP binding (GO:0005524), and protein serine/threonine kinase activity (GO:0004674). Additionally, in the CC category, the GO terms were related to different cellular components including plasma membrane (GO:0005886), cytoplasm (GO:0005737), and nucleus (GO:0005634), which was consistent with the previous results of subcellular localization prediction of PKs.

### Segmental duplication events among the pecan kinome

Gene duplications functioned in the expansion of the pecan kinome, and the gene copies generated by duplication contributed largely to the evolution of novel functions and environmental adaptation^[16]^. Segmental duplications occur frequently in higher plants since most of them are diploidized polyploids, and retain multiple duplicated chromosomal blocks within the genomes^[17]^. The segmental duplication events in the pecan kinome were investigated using MCScanX, and 169 duplication events with 285 PK genes from 63 subfamilies were found, 29.47% of PK members were evolved by segmental duplication, suggesting segmental duplication contributed to the expansion of the pecan kinome (Supplemental Table S6). Among the 285 PK genes, 145 were RLK group members, indicating 25.66% of the RLK group members resulted from segmental duplication. Moreover, 58.82% and 44.68% of the CK1 and CAMK group members resulted from segmental duplication, respectively.

**Figure 4:**
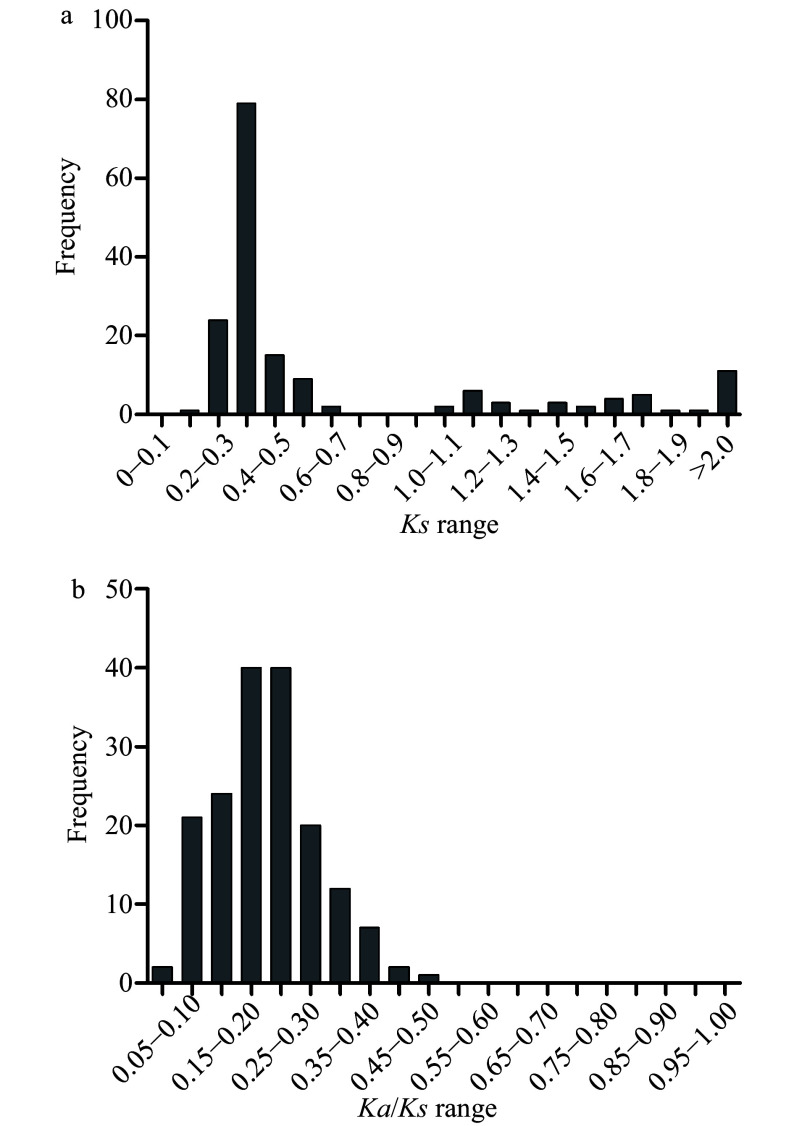
Distribution of relative *Ks* (a), and *Ka/Ks* (b) frequency among segmental duplication events in the pecan kinome.

The non-synonymous substitutions (*Ka*), and synonymous substitutions (*Ks*) of the 169 duplication events were calculated, and *Ks* was applied as a time indicator to evaluate the relative date of duplication blocks (Supplemental Table S6). Among the segmental duplication events in the pecan kinome, the distribution of *Ks* values showed that the *Ks* ranged from 0.2 to 3.6, and peaked at 0.3 to 0.4 (Fig. 4a). Intriguingly, 70.41% of the frequency of *Ks* values were less than 0.5, indicating recent duplications played an important role in the expansion of the pecan kinome. The *Ka*/*Ks* values of segmentally duplicated gene pairs were further analyzed to determine the selection pressures influencing sequence divergence. A value of *Ka*/*Ks* > 1 represents positive selection, a value of *Ka*/*Ks* < 1 represents negative selection, whereas a value of *Ka*/*Ks* = 1 indicates neutral selection. Among these duplication events tested, the *Ka*/*Ks* values ranged from 0.043 to 0.46, suggesting these PK genes have experienced strong negative selection (Fig. 4b).

### Expression analysis of pecan PK genes during embryo development

The kernels of pecan nuts are nutritious with a high economic value. To investigate the expression patterns of PK genes in the developing pecan embryo, the expression data of 967 pecan PK genes through three typical stages, PEY1 (early stage), PEY2 (stage with fully extended cotyledons) and PEY3 (fully matured stage) of embryonic development were retrieved (Supplemental Table S7)^[15]^. According to the hierarchical clustering results of PK genes during embryo development, we found that genes in different PK subfamilies commonly performed various expression patterns (Supplemental Fig. S3). About one-third of PK genes showed very low expression levels in all three stages, such as *CIL1226S0040* (CAMK_CDPK), *CIL0942S0004* (STE_STE11), *CIL1211S0038* (WNK_NRBP), and *CIL0895S0070* (RLK-Pelle_DLSV). We also found the majority of the PK genes with low expression levels among pecan embryo development stages were the RLK group members. In contrast, some genes including *CIL1119S0056*, *CIL0940S0189*, *CIL1575S0001*, *CIL1611S0012* in the CAMK group and *CIL0902S0027*, *CIL0893S0285*, *CIL1032S0081* in the CMGC group showed high expression levels in all stages tested. Many other PK genes performed specific expression patterns in different stages.

According to the expression levels of PK genes during pecan embryo development, we found the PK genes performed various expression patterns. These genes were then divided into eight clusters based on the expression patterns at three stages during embryo development (Supplemental Fig. S4), and three major clusters (cluster 0, 1, and 3) contained more than 100 members (Supplemental Table S8). Cluster 0 had the most PK genes (222) among the eight clusters, and the expression levels of genes in cluster 0 were gradually decreased during embryo development, however, 14 PK genes in cluster 7 were gradually increased.

**Figure 5:**
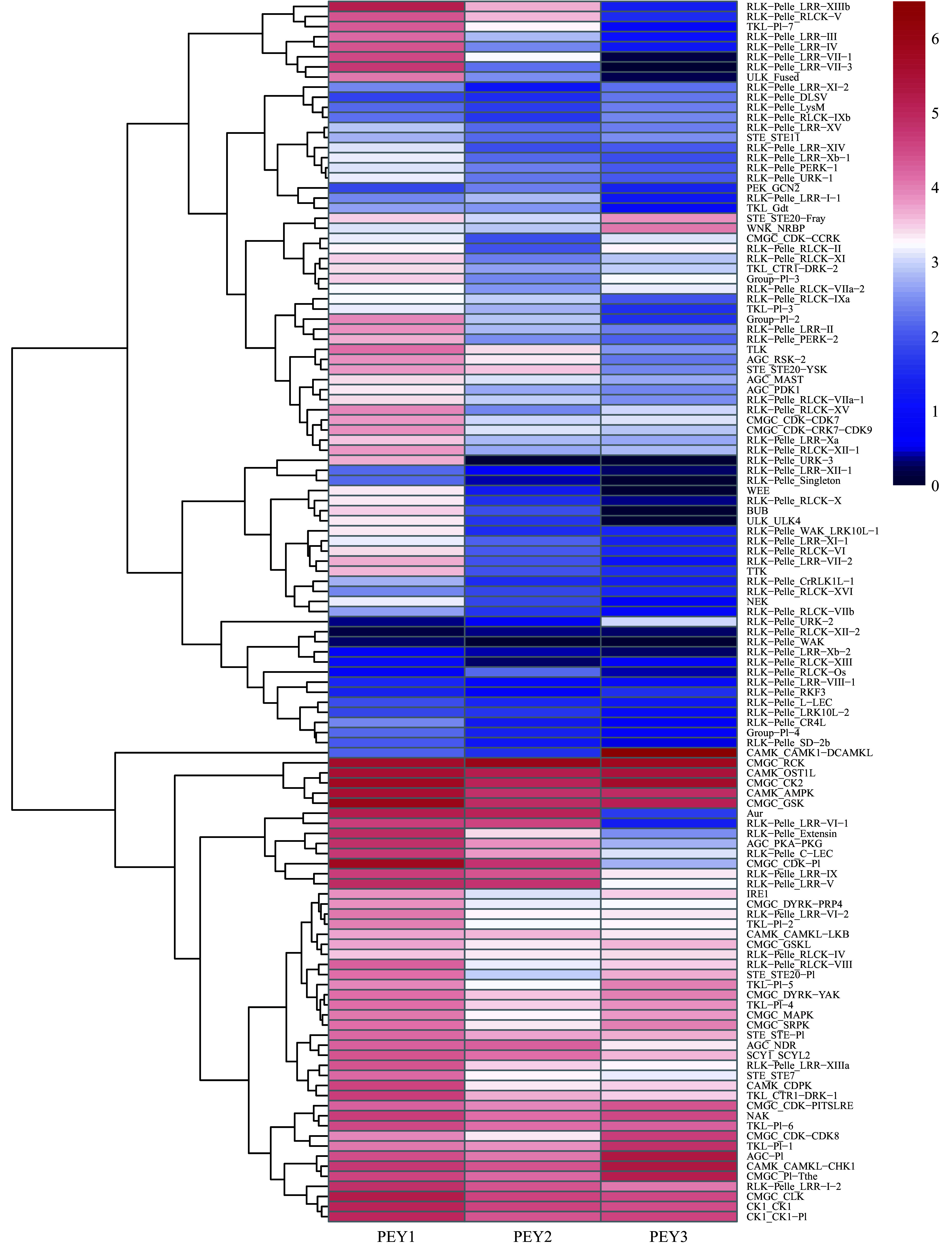
Expression analysis of the pecan PK subfamilies during embryo development. The expression data of different PK subfamilies collected from three representative periods of embryonic development of pecan including the early period (PEY1), the period with fully extended cotyledons (PEY2), and the fully matured period of the embryos (PEY3). Log_2_ (FPKM+1) values were performed according to the red-white-blue color scale. The heatmap was generated using the R package pheatmap with hierarchical clustering.

To further investigate the relationship between different pecan PK families during embryo development, the expression data of PK genes in each subfamily were averaged and a heatmap with clustering analysis was created (Fig. 5). According to the expression analysis at the subfamily level, these 121 PK gene subfamilies performed distinct expression patterns during embryo development. Some subfamilies such as CAMK_OST1L, CAMK_AMPK, CMGC_RCK, CMGC_CK2 showed high expression levels in all three representative stages. In contrast, several subfamilies in the RLK group including RLK-Pelle_RLCK-XII-2, RLK-Pelle_WAK, RLK-Pelle_LRR-Xb-2, RLK-Pelle_XIII presented low expression levels, which is consistent with previous results (Supplemental Fig. S3). Surprisingly, the remaining subfamilies in the RLK group were highly expressed in the PEY1 stage and down-regulated, indicating they might negatively regulate the embryo development process.

### Expression and co-expression analysis of pecan PK genes under drought stress

Protein kinases commonly play essential roles in response to environmental stresses including drought stress^[10, 18, 19]^. In order to explore the expression levels of pecan PK genes under drought stress, the RNA-Seq data were retrieved with FPKM values. In total, the expression data of 952 available genes in response to drought treatment were collected (Supplemental Table S9). The results indicated that PK subfamilies showed different expression patterns in response to drought (Supplemental Fig. S5). About half of the subfamilies in the RLK group exhibited low expression levels. By contrast, most subfamilies in AGC, CAMK, CMGC, CK1, and TKL groups were highly expressed, indicating these subfamily members may play essential roles in response to drought stress.

**Figure 6:**
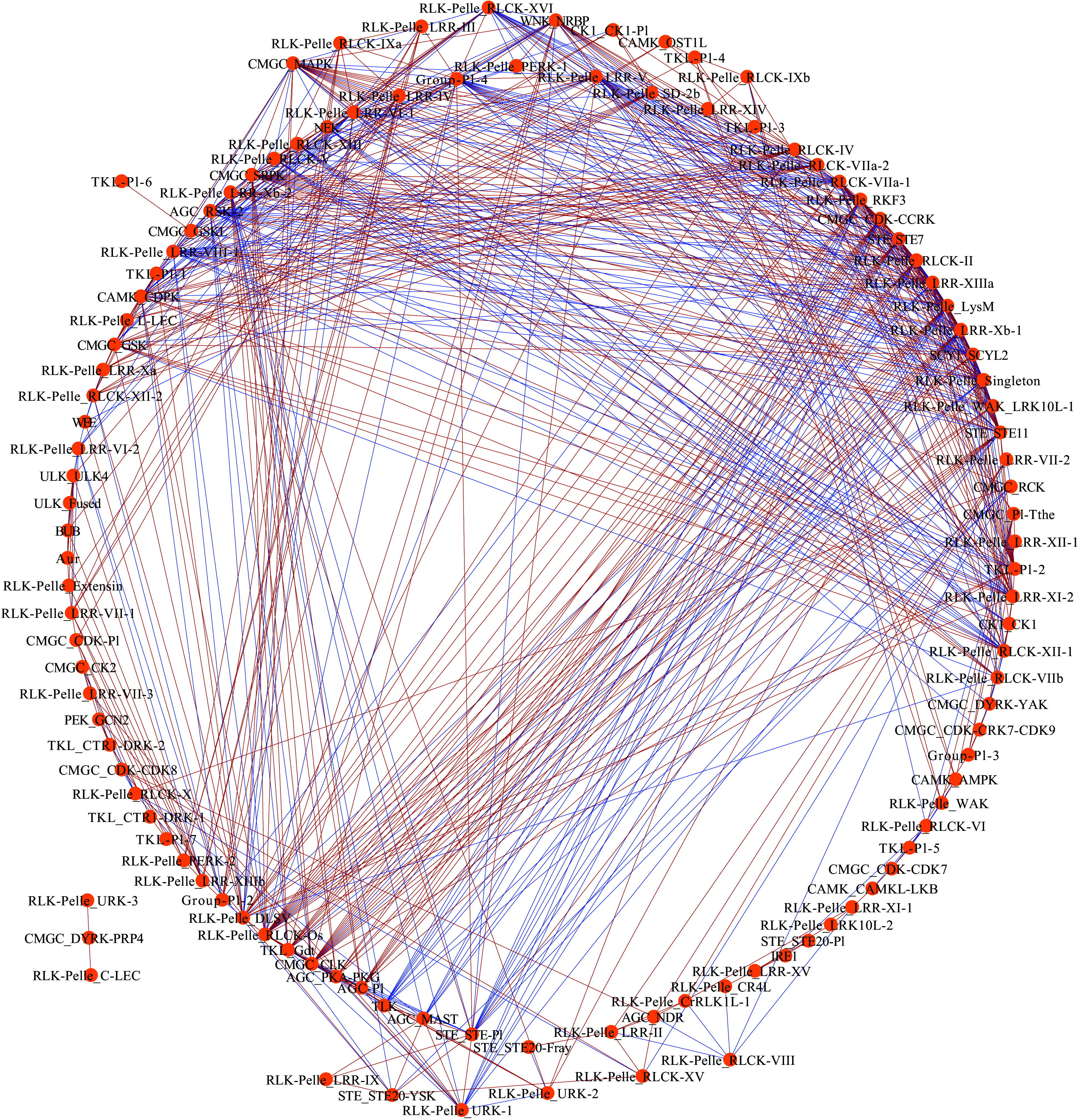
Co-expression networks of pecan PK subfamilies in response to drought. Each node indicated pecan PK subfamilies, and the edges indicated significant co-expression between subfamilies with a PCC of at least 0.9 (*p* < 0.01). Blue-colored edges indicate negative correlations, and red-colored edges indicate positive correlations.

To investigate the mutual relationships between pecan PK subfamilies under drought treatment, the co-expression networks were constructed (Fig. 6). The networks contained 112 nodes (PK subfamilies) and 690 edges (co-expression events) with one main network and one subnetwork. The main network had 109 nodes and 688 edges, and each node harbored a different number of edges varying from 1 to 31. Among these PK subfamilies, 30 had more than 20 edges, four subfamilies including AGC_RSK-2, CMGC_MAPK, RLK-Pelle_LRR-XIIIa and RLK-Pelle_RLCK-II had the maximum number of regulatory edges, and were considered as central hubs in the co-expression networks. According to the co-expression events, 421 showed significantly positive correlations, while the remaining 269 showed significantly negative correlations.

**Figure 7:**
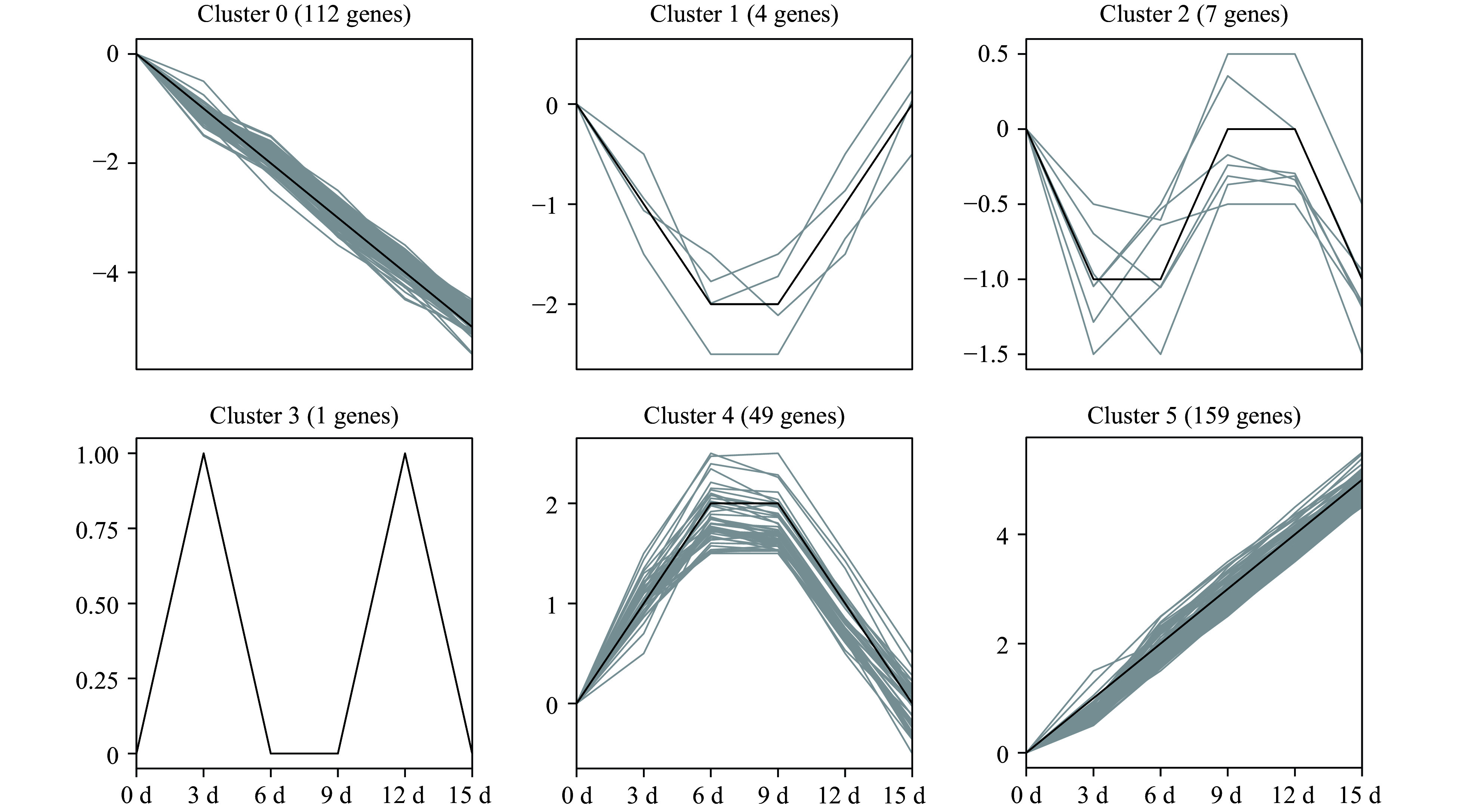
Temporal changes of differentially expressed PK genes under drought stress in pecan. One-year-old grafted 'Pawnee' trees were subjected to drought by withholding water for 0, 3, 6, 9, 12, and 15 d. Leaf samples were collected and used for RNA-Seq experiments. Expression data of 332 DEGs were retrieved and clustered into six clusters. The PK genes of each cluster are also listed in Supplemental Table S10.

In total, 589 PK genes in the pecan kinome were identified as differentially expressed genes (DEGs) (| log_2 _(fold change) | ≥ 1, FDR < 0.05). Among them, 257 having a FPKM value < 10 at all time points were considered as lowly expressed genes; 332 PK genes had a FPKM value ≥ 10 in at least one time point. To analyze the expression patterns of pecan PK genes in response to drought stress, the 332 DEGs were further divided into clusters after filtering the lowly expressed PK genes (Fig. 7). Six different clusters with similar expression patterns were performed and members in different clusters ranged from 1 (cluster 3) to 159 (cluster 5), the detailed PK genes in each cluster are shown in Supplemental Table S10. Cluster 5 contained the most numbers of PK genes and the expression levels were gradually increasing under drought stress, however, genes in cluster 0 were gradually decreasing. Interestingly, some DEGs in a subfamily showed similar expression patterns. For example, eight genes in CAMK_CAMKL-CHK1, 11 genes in CAMK_CDPK, 22 genes in RLK-Pelle_DLSV, and seven members in STE_STE11 were all gradually up-regulated in cluster 5, suggesting these PK subfamily members might function in response to drought stress.

## DISCUSSION

Reversible phosphorylation, performed by PKs, is one of the most crucial post-translational modifications, and involved in multiple cellular processes^[19, 20]^. Although functional analysis of some PKs has been discovered in model plants including *Arabidopsis* and rice^[21−23]^, few PKs have been well understood in woody plants due to limited genome information. The recent release of the *Carya illinoinensis* genome sequence, an economically important nut tree cultivated worldwide, provides the chance to characterize and understand the regulatory networks of the pecan kinome. In the present research, 967 putative pecan PKs were identified using bioinformatic methods (Supplemental Fig. S1), which accounted for 3.11% (967/31,075) of protein-coding genes in the pecan genome^[15]^. This proportion of PKs in pecan was lower than that in soybean (4.7%), rice (4.1%), maize (3.8%), and *Arabidopsis* (3.4%), while higher than that of pineapple (2.8%)^[6, 7, 24, 25]^. The classification of PKs from 25 plant species showed that gene numbers ranged from 326 to 2535, and the kinome size was significantly larger in the flowering plants, while two algae species including *Chlamydomonas reinhardtii* and *Volvox carteri* had 503 and 326 PKs, respectively^[6]^. *Ostreococcus tauri*, a unicellular species of green alga, only possessed 133 PKs in its genome, amounting to 1.7%^[26]^.

Plant kinomes were commonly categorized into different groups and families based on the sequence difference of the kinase domain. The pecan kinome was divided into 20 different groups, and the RLK group was found to be the largest, containing more than half of the members (565) in the pecan kinome (Fig. 1), a similar phenomenon was also found in other flowering plants including *Arabidopsis*, grapevine, and rice (Supplemental Fig. S2)^[6, 8, 27]^. Interestingly, *Chlamydomonas reinhardtii* and *Volvox carteri* contained only two and three members in the RLK group, respectively. The large numbers of PK genes in flowering plants can be mainly attributed to the dramatic expansion of a few PK groups, especially the RLK group^[28]^. The number of subfamilies in the pecan kinome (121) was larger than that in pineapple (116), and similar to the soybean kinome (122)^[7, 25]^.

Duplication contributes to the evolution of novel gene functions including stress adaptation, disease resistance, and also makes major contributions to the large size of the RLK group in higher plants^[16]^. Over 90% of the increase in regulatory genes was caused by gene duplication in the *Arabidopsis* lineage^[29]^. In the pecan kinome, 29.47% (285/967) of the PK genes with 169 gene pairs were generated from segmental duplication, 145 of them were RLK genes and separated into 34 subfamilies (Supplemental Table S6), 10,530 paralogous pairs were found in the pecan genome^[15]^. Different families in the RLK group showed various expansion patterns, large families such as LRR and RLCK make important contributions to the expansion of the large size of the RLK group. Sixty-five and 49 PKs in LRR and RLCK families were generated from gene duplication, respectively, which is consistent with the previous results found in soybean^[7]^. The distribution of *Ks* values can be used to estimate the evolutionary date, more than 70% of duplicated genes in the pecan kinome occurred more recently (Fig. 4a). The ratio of *Ka*/*Ks* was commonly used to detect the history of selection pressure on coding sequences of duplicated genes^[30]^. In this study, *Ka*/*Ks* values of the 169 duplication events in the pecan kinome were less than 0.05, strong negative selection drove the evolution of the PKs in pecan (Fig. 4b). In a previous study, negative selection was also found to be the primary influence on PK genes in pineapple, negative selection indicated the process of removing deleterious mutations^[31]^.

PKs were generally related to the transmission of extracellular signals to the nucleus by activating or repressing target proteins, and subcellular localization information of PKs might help to explain protein's function^[32]^. We predicted the subcellular localization data of PKs in different groups, and about half of the RLK group members were located in the plasma membrane (Fig. 2), however, only 7% of PKs in RLCK families were membrane-located due to the absence of extracellular ligand-binding domains^[33]^. PKs in the non-RLK clade showed different subcellular localization features, such as most AGC group members were nucleus-located and more than 70% of CAMK group members were localized in the cytoplasm, similar results were also found in the pineapple kinome^[25]^.

Plant PKs, especially calcium-dependent protein kinases (CDPKs), mitogen-activated protein kinase (MAPK) cascades, sucrose non-fermenting1-related protein kinases (SnRKs), and RLKs have been well investigated and functionally analyzed in some model plants and crops^[18, 19, 33]^. To find the key genes more efficiently from the rice kinome with thousands of members, the rice kinase database (RKD) with PK genes in various tissues, under abiotic and biotic stresses was built^[34]^. However, limited expression information of PK genes is available for pecan. Expression levels might provide evidence of gene function, then RNA-Seq data of pecan PK genes were analyzed to obtain the central candidates during embryo development or response to drought stress. The expression patterns of pecan PK subfamilies during embryo development revealed many RLK subfamilies were down-regulated, especially some LRR subfamilies (Fig. 5), and this family has been found to play a role in embryo formation^[35, 36]^.

Drought stress could seriously impact food and energy security, and PKs have key functions in response to abiotic stresses including drought^[21]^. The expression data of PK subfamilies in pecan were analyzed under drought stress, while half of the RLK subfamilies performed low expression levels (Supplemental Fig. S5), these subfamilies also showed low expression in soybean and grapevine in response to drought^[7, 8]^. Furthermore, the differentially expressed genes in the pecan kinome were selected and divided into six clusters based on their different expression patterns, Cluster 5 contained 159 PK genes which were increased under drought stress, including three subfamilies such as CAMK_CAMKL-CHK1, CAMK_CDPK, and CAMK_OST1L in the CAMK group (Fig. 7). The CAMK_CAMKL-CHK1 subfamily, known as CBL-interacting protein kinase (CIPK), was involved in the drought stress response^[21]^. *MdCIPK6L* was up-regulated under drought stress, and the overexpression plants remarkably enhanced the tolerance to drought stress^[37]^. CcCBL1-CcCIPK14 module positively regulated drought tolerance via enhancing flavonoid biosynthesis in pigeon pea^[38]^. *NtCIPK11* was up-regulated significantly in *Nitraria tangutorum* after mannitol treatment, and overexpression lines in *Arabidopsis* improved both drought and salt tolerance^[39]^. CDPK and CAMK_OST1L (named as SnRK2) genes have been proved to function in plant drought stress response^[19, 40]^. Among the 159 members in Cluster 5, 95 were RLK group genes and distributed in 28 subfamilies, which accounted for 59.75% (Supplemental Table S10). The receptor-like kinases activate the downstream signaling pathway via perceiving the extracellular signals and phosphorylating the targets, and drought stress caused the most notable effect on rice RLKs^[41]^. Intriguingly, nearly one-third of the genes in the largest subfamily, RLK-Pelle_DLSV, were found in Cluster 5, indicating this subfamily may play a key role in response to drought stress.

## CONCLUSIONS

Plant protein kinases are important regulators of a variety of cellular processes including plant development and stress responses. In this study, a total of 967 PKs were annotated in the pecan genome, and divided into 121 subfamilies with 20 groups. Gene duplication functioned in the expansion of the pecan kinome, and the segmentally duplicated events suffered strong negative selection based on the *Ka*/*Ks* ratios. Moreover, different PK subfamilies in the pecan kinome performed dynamic transcript abundance during embryo development. In addition, pecan PK genes presented various expression patterns in response to drought, and most of them were differentially expressed. This research provides valuable information concerning pecan PKs, and lays a good foundation for further functional investigation of these genes during embryo development and drought stress responses.

## MATERIALS AND METHODS

### Computational retrieval and classification of pecan PKs

All pecan protein and nucleotide sequences were downloaded from the GigaScience database (http://gigadb.org/dataset/100571)^[15]^. To uncover all the protein kinases in the pecan genome, Hidden Markov Models (HMMs) of the protein kinase clan including Pkinase (PF00069) and Pkinase_Tyr (PF07714) were downloaded from the Pfam website (http://pfam.xfam.org)^[42]^. HMMER software version 3.1b2 was used to investigate putative PKs, with an e-value cutoff of 1e-5^[43]^. Each candidate PK gene was further verified with the existence of the kinase domain using SMART (http://smart.embl-heidelberg.de)^[44]^. The putative PK was considered as a typical protein kinase if the domain alignments covered at least 50% of the kinase domain models^[6]^.

All identified protein kinases in *Carya illinoinensis* (pecan kinome) were classified into various groups, families, and subfamilies by HMMs constructed from a previous classification of 25 plant kinomes^[6]^.

### Sequence alignment and phylogenetic analysis

The kinase catalytic domain sequences of pecan PK proteins were retrieved using a perl script. Multiple sequence alignment was performed using MAFFT version 7 with the G-INS-I strategy (https://mafft.cbrc.jp/alignment/software)^[45]^. A Maximum-likelihood tree was constructed with the domain sequences using FastTree version 2.1 with the default setting (http://www.microbesonline.org/fasttree) to verify pecan kinome classification results^[46]^.

### In silico analysis of pecan PK sequences

Physical properties of the pecan PK proteins including molecular weight (MW), isoelectric points (pIs), and grand average of hydropathicity (GRAVY) were collected using online ExPASy ProtParam server (https://web.expasy.org/protparam).

### Subcellular localization prediction

To investigate the potential function of PKs in various cellular organelles, protein subcellular localization was predicted using CELLO v2.5 (http://cello.life.nctu.edu.tw)^[47]^.

### Intron numbers and domain organizations

Intron numbers of all pecan PK genes were collected from the General Feature Format (gff) file from the GigaDB^[15]^. To analyze the domain organization patterns of the PKs, the Pfam database was used to identify the conserved domains according to the protein sequences of PKs with an e-value threshold of 1e-5.

### GO functional analysis of pecan PKs

OmicsBox software version 1.4 (https://www.biobam.com/omicsbox) was applied to analyze the Gene Ontology (GO) functional information. The annotations of GO terms were collected from Gene Ontology Consortium (http://geneontology.org).

### Segmental duplication events identified in the pecan kinome

All of the pecan PK sequences were searched against themselves by NCBI-BLAST 2.7.1+^[48]^. Then, segmental duplication events within the pecan kinome were investigated using Multiple Collinearity Scan toolkit (MCScanX) according to the manual^[49]^.

### Estimation of the *Ka* and *Ks* values

The coding sequences (CDS) of the PK genes of duplication events were aligned with ClustalW^[50]^. To investigate the selection pressure of duplicate events, the non-synonymous substitutions (*Ka*) and synonymous substitutions (*Ks*) were calculated using TBtools software version 1.0971^[51]^. *Ks* values were further used to determine the date of duplication events, and the *Ka*/*Ks* ratios revealed the selection pressure of duplication events^[25]^.

### Transcriptome analysis of PK genes during pecan embryo development

Publicly available transcriptome datasets were used to investigate the expression patterns of kinase genes in pecan. The expression data of PK genes in three key stages during embryo development in cultivar 'Pawnee' were retrieved with FPKM values (fragments per kilobase per million of reads mapped) from the NCBI database (BioProject number: PRJNA435846)^[15]^.

### Plant materials, growth conditions, and sample collection

One-year-old pecan seedlings, propagated from seeds (collected from 'Pawnee' trees in October) were selected as rootstock, and the genome sequenced cultivar 'Pawnee' was used as scion. Patch budding was selected and used for pecan grafting in August. After 12 months, the grafted plants were moved to a growth chamber with 14 h light at 24 °C/10 h dark at 22 °C photoperiods. The grafted plants were grown in pots under well-watered conditions for 30 d, then water was withheld for 15 d. On each grafted plant, a single compound leaf from the top was selected, and the second set of leaflets from the apex of this compound leaf were collected. Plant leaf samples were harvested at 0, 3, 6, 9, 12, and 15 d after drought treatment. The harvested samples were frozen in liquid nitrogen immediately, then stored at −70 °C to prevent RNA degradation until RNA isolation was carried out.

### RNA isolation and RNA-Seq analyses of pecan PKs

Three biological replicates of pecan leaf samples under drought treatment were harvested and applied for RNA-Seq experiments, and each biological replicate was collected from at least three grafted plants. Total RNA was isolated using Trizol reagent (Invitrogen, Carlsbad, USA) following the manufacturer's instructions. RNA quality was detected using RNase-free agarose gel electrophoresis and NanoDrop 2000 spectrophotometer (Thermo Fisher Scientific, Wilmington, USA). Totally, 1 μg RNA per sample was reverse transcribed to cDNA, and cDNA libraries were sequenced using an Illumina Novaseq 6000 platform (GeneDenovo, Guangzhou, China).

Reference-guided mapping was performed with the latest genome assembly of pecan^[15]^. The index of the pecan reference genome was built, and clean reads were aligned to the pecan reference genome using HISAT2.2.4^[52]^. The mapped reads were assembled using StringTie version 1.3.1 in a reference-based method^[53]^. The expression abundance of pecan PK genes was quantified by calculating the FPKM value using RSEM software^[54]^. The raw data of RNA-Seq have been deposited and made available in NCBI with the accession number GSE179336.

Differential expression analysis of RNA-Seq data between the control (0 d) and drought-treated datasets at 3, 6, 9, 12, and 15 d was presented by DESeq2 software^[55]^, the PK genes with the parameter of FDR (false discovery rate) < 0.05 and the absolute value of log_2_ Ratio ≥ 1 were considered as differentially expressed genes.

### Expression patterns of PK genes in pecan

Genes in the pecan kinome were classified into different clusters based on their expression patterns (*p* < 0.05) using the Short Time-series Expression Miner (STEM) software (http://www.cs.cmu.edu/~jernst/stem)^[56]^.

### Co-expression networks of pecan kinase subfamilies

To investigate the topological relationships between pecan PK subfamilies, the co-expression networks were constructed using the Pearson correlation coefficient (PCC) based on the expression profile of pecan PK genes during drought stress response using IBM SPSS software version 25 (https://www.ibm.com/products/spss-statistics). All of the gene expression data of PKs in each subfamily were averaged, and the subfamily pairs with absolute values of PCC higher than 0.9 were retrieved at the 0.01 significance level (*p*-value) and used for co-expression network analysis. The networks were eventually visualized using Cytoscape software version 3.7.1 (https://cytoscape.org)^[57]^.

## SUPPLEMENTARY DATA

Supplementary data to this article can be found online.
