# Supplementary material for: Pecan kinome: classification and expression analysis of all protein kinases in Carya illinoinensis
Source: For Res (Fayettev). 2021 Aug 18;1:14. doi: 10.48130/FR-2021-0014 (PMC11524300; doi:10.48130/FR-2021-0014)
Supplement: Supplementary file 1 — Supplementary data to this article can be found online. [file FR-2021-0014-S1.zip › 10.48130_FR-2021-0014-Suppl-FigureS5.docx]

**Pecan kinome: classification and expression analysis of all protein kinases in *Carya illinoinensis***

Kaikai Zhu1, Pinghua Fan1, Hui Liu2, Juan Zhao1, Pengpeng Tan1, Zhenghai Mo3 and Fangren Peng1*

*1 Co-Innovation Center for Sustainable Forestry in Southern China, Nanjing Forestry University, Nanjing, Jiangsu 210037, China*

*2 State Key Laboratory of Crop Genetics and Germplasm Enhancement, Ministry of Agriculture and Rural Affairs Key Laboratory of Biology and Germplasm Enhancement of Horticultural Crops in East China, College of Horticulture, Nanjing Agricultural University, Nanjing, Jiangsu 210095, China*

*3 Institute of Botany, Jiangsu Province and Chinese Academy of Sciences, Nanjing 210014, China*

* Corresponding author, E-mail: [frpeng@njfu.edu.cn](mailto:frpeng@njfu.edu.cn)

**Supplementary Data**

Supplementary data associated with this article can be found, in the online version.

Supplemental Table S1: Kinase domain annotation of 967 pecan PKs.

Supplemental Table S2: Family classification of pecan PKs with related information.

Supplemental Table S3: Domain organization of pecan PKs.

Supplemental Table S4: List of pecan protein kinases containing multiple kinase domains.

Supplemental Table S5: GO IDs of pecan PKs.

Supplemental Table S6: Segmental duplication events and *Ka/Ks* values of pecan protein kinases.

Supplemental Table S7: FPKM values of pecan PK genes during embryo development.

Supplemental Table S8: Genes in eight groups with different expression patterns during pecan embryo development.

Supplemental Table S9: Average FPKM expression values of pecan PK genes under drought stress.

Supplemental Table S10: Differentially expressed PK genes in six clusters with different expression patterns under drought stress.

Supplemental Fig. S1: Phylogenetic classification of pecan PKs.

Supplemental Fig. S2: Phylogenetic classification of PKs in four different species.

Supplemental Fig. S3: Expression of PK genes during embryo development in pecan.

Supplemental Fig. S4: Different expression patterns of pecan PK genes during embryo development.

Supplemental Fig. S5: Expression data of PK subfamilies with drought treatment in pecan.

**Supplemental Fig. S1** Phylogenetic classification of pecan PKs. The phylogenetic tree was generated with amino sequences of the kinase domain with maximum-likelihood method. PK subfamilies were highlighted with different colors.

**Supplemental Fig. S2** Phylogenetic classification of PKs in four different species. The phylogenetic tree was generated with amino sequences of the kinase domain from four different species (967 from pecan, 1006 from *Arabidopsis*, 1168 from grape and 758 from pineapple) with maximum-likelihood method.

**Supplemental Fig. S3** Expression of PK genes during embryo development in pecan. Log2 (FPKM+1) values were performed according to the red-white-blue color scale, and the heatmaps were generated using the R language with hierarchical clustering.

**Supplemental Fig. S5**

**Supplemental Fig. S5** Expression data of PK subfamilies with drought treatment in pecan. Log2 (FPKM+1) values were performed according to the red-white-blue color scale, and the heatmaps were generated using R language with hierarchical clustering.
