# Supplementary figures and images for: Pecan kinome: classification and expression analysis of all protein kinases in Carya illinoinensis
Source: For Res (Fayettev). 2021 Aug 18;1:14. doi: 10.48130/FR-2021-0014 (PMC11524300; doi:10.48130/FR-2021-0014)

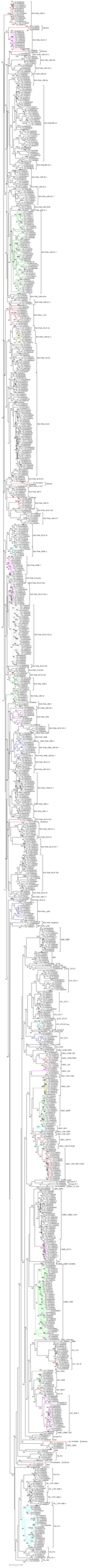

Supplement: Supplementary file 1 — Supplementary data to this article can be found online. [file FR-2021-0014-S1.zip › 10.48130_FR-2021-0014-Suppl-FigureS1.pdf]

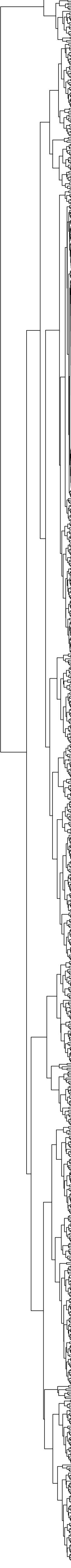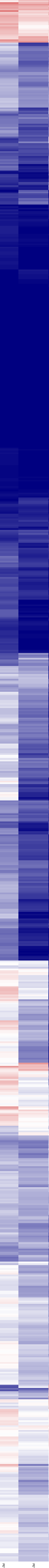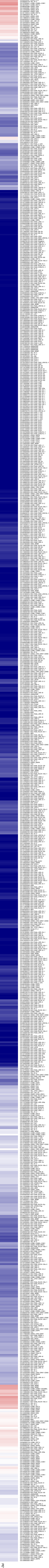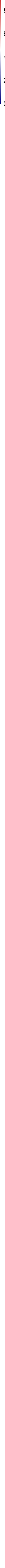

Supplement: Supplementary file 1 — Supplementary data to this article can be found online. [file FR-2021-0014-S1.zip › 10.48130_FR-2021-0014-Suppl-FigureS3.pdf]
