# Supplementary material for: Pecan kinome: classification and expression analysis of all protein kinases in Carya illinoinensis
Source: For Res (Fayettev). 2021 Aug 18;1:14. doi: 10.48130/FR-2021-0014 (PMC11524300; doi:10.48130/FR-2021-0014)
Supplement: Supplementary file 1 — Supplementary data to this article can be found online. [file 10.48130_FR-2021-0014-Suppl-FigureS2.pdf › 10.48130_FR-2021-0014-Suppl-FigureS2.pdf]

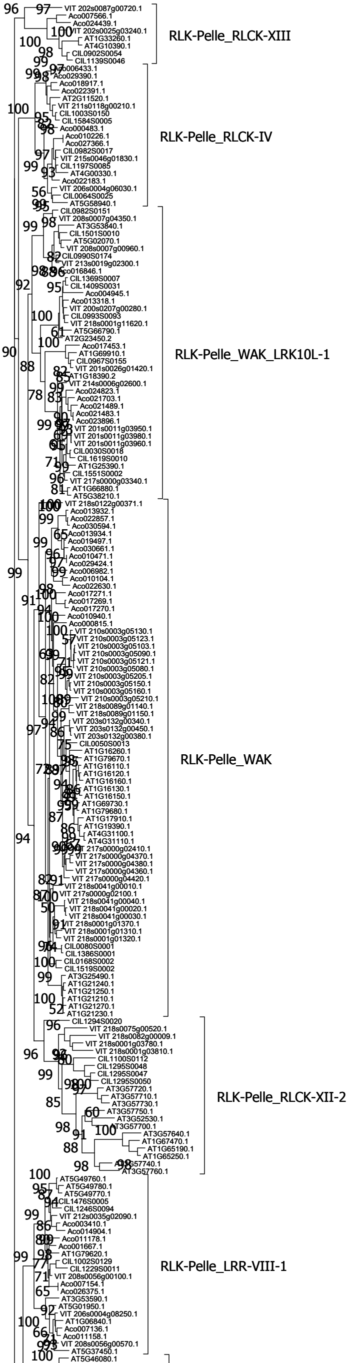
RLK-Pelle_RLCK-XIII
RLK-Pelle_RLCK-IV
RLK-Pelle_WAK_LRK10L-1
RLK-Pelle_WAK
RLK-Pelle_RLCK-XII-2
RLK-Pelle_LRR-VIII-1
VIT 202s0087g00720.1
Aco007566.1
Aco024439.1
VIT 202s0025g03240.1
AT1G33260.1
AT4G10390.1
CIL0902S0054
CIL1139S0046
Aco029390.1
Aco018917.1
Aco022391.1
AT2G11520.1
VIT 211s0118g00210.1
CIL1003S0150
CIL1584S0005
Aco000483.1
Aco010226.1
Aco027366.1
CIL0982S0017
VIT 215s0046g01830.1
CIL1197S0085
AT4G00330.1
Aco022183.1
VIT 206s0004g06030.1
CIL0064S0025
AT5G58940.1
CIL0982S0151
VIT 208s0007g04350.1
AT3G53840.1
CIL1501S0010
AT5G02070.1
VIT 208s0007g00960.1
CIL0990S0174
VIT 213s0019g02300.1
CIL1369S0007
CIL1409S0031
Aco004945.1
Aco013318.1
VIT 200s0207g00280.1
CIL0993S0093
VIT 218s0001g11620.1
AT5G66790.1
AT2G23450.2
Aco017453.1
AT1G69910.1
CIL0967S0155
VIT 201s0026g01420.1
AT1G18390.2
VIT 214s0006g02600.1
Aco024823.1
Aco021703.1
Aco021489.1
Aco021483.1
Aco023896.1
VIT 201s0011g03950.1
VIT 201s0011g03980.1
VIT 201s0011g03960.1
CIL0030S0018
CIL1619S0010
AT1G25390.1
CIL1551S0002
VIT 217s0000g03340.1
AT1G66880.1
AT5G38210.1
VIT 218s0122g00371.1
Aco013932.1
Aco022857.1
Aco030594.1
Aco013934.1
Aco019497.1
Aco030661.1
Aco010471.1
Aco029424.1
Aco006982.1
Aco010104.1
Aco022630.1
Aco017271.1
Aco017269.1
Aco017270.1
Aco010940.1
Aco000815.1
VIT 210s0003g05130.1
VIT 210s0003g05123.1
VIT 210s0003g05103.1
VIT 210s0003g05090.1
VIT 210s0003g05121.1
VIT 210s0003g05080.1
VIT 210s0003g05205.1
VIT 210s0003g05150.1
VIT 210s0003g05160.1
VIT 210s0003g05210.1
VIT 218s0089g01140.1
VIT 218s0089g01150.1
VIT 203s0132g00340.1
VIT 203s0132g00450.1
VIT 203s0132g00380.1
CIL0050S0013
AT1G16260.1
AT1G79670.1
AT1G16110.1
AT1G16120.1
AT1G16160.1
AT1G16130.1
AT1G16150.1
AT1G69730.1
AT1G79680.1
AT1G17910.1
AT1G19390.1
AT4G31100.1
AT4G31110.1
VIT 217s0000g02410.1
VIT 217s0000g04370.1
VIT 217s0000g04380.1
VIT 217s0000g04360.1
VIT 217s0000g04420.1
VIT 218s0041g00010.1
VIT 217s0000g02100.1
VIT 218s0041g00040.1
VIT 218s0041g00020.1
VIT 218s0041g00030.1
VIT 218s0001g01370.1
VIT 218s0001g01310.1
VIT 218s0001g01320.1
CIL0080S0001
CIL1386S0001
CIL0168S0002
CIL1519S0002
AT3G25490.1
AT1G21240.1
AT1G21250.1
AT1G21210.1
AT1G21270.1
AT1G21230.1
CIL1294S0020
VIT 218s0075g00520.1
VIT 218s0082g00009.1
VIT 218s0001g03780.1
VIT 218s0001g03810.1
CIL1100S0112
CIL1295S0048
CIL1295S0047
CIL1295S0050
AT3G57720.1
AT3G57710.1
AT3G57730.1
AT3G57750.1
AT3G52530.1
AT3G57700.1
AT3G57640.1
AT1G67470.1
AT1G65190.1
AT1G65250.1
AT3G57740.1
AT3G57760.1
AT5G49760.1
AT5G49780.1
AT5G49770.1
CIL1476S0005
CIL1246S0094
VIT 212s0035g02090.1
Aco003410.1
Aco014904.1
Aco011178.1
Aco001667.1
AT1G79620.1
CIL1002S0129
CIL1229S0011
VIT 208s0056g00100.1
Aco007154.1
Aco026375.1
AT3G53590.1
AT5G01950.1
VIT 206s0004g08250.1
AT1G06840.1
Aco007136.1
Aco011158.1
VIT 208s0056g00570.1
AT5G37450.1
AT5G46080.1

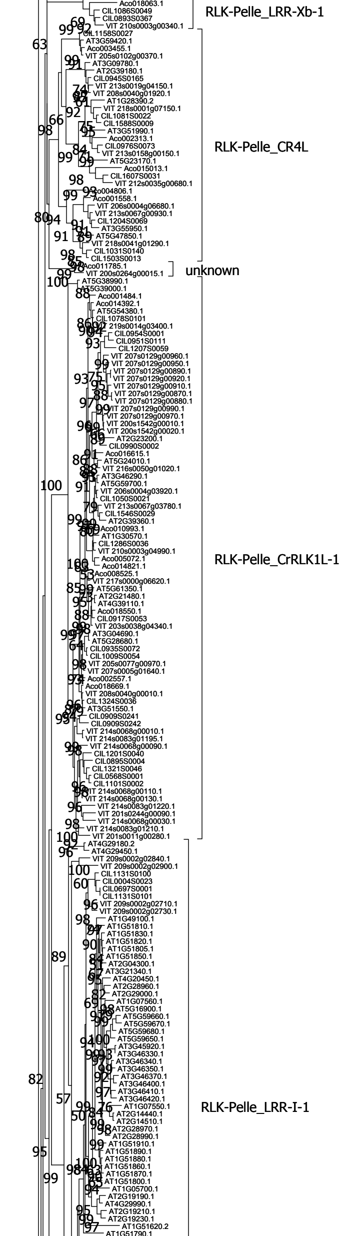
RLK-Pelle_LRR-Xb-1
RLK-Pelle_CR4L
unknown
RLK-Pelle_CrRLK1L-1
RLK-Pelle_LRR-I-1
Aco018063.1
CIL1086S0049
CIL0893S0367
VIT 210s0003g00340.1
CIL1158S0027
AT3G59420.1
Aco003455.1
VIT 205s0102g00370.1
AT3G09780.1
AT2G39180.1
CIL0945S0165
VIT 213s0019g04150.1
VIT 208s0040g01920.1
AT1G28390.2
VIT 218s0001g07150.1
CIL1081S0022
CIL1588S0009
AT3G51990.1
Aco002313.1
CIL0976S0073
VIT 213s0158g00150.1
AT5G23170.1
Aco015013.1
CIL1607S0031
VIT 212s0035g00680.1
Aco004806.1
Aco001558.1
VIT 206s0004g06680.1
VIT 213s0067g00930.1
CIL1204S0069
AT3G55950.1
AT5G47850.1
VIT 218s0041g01290.1
CIL1031S0140
CIL1503S0013
Aco011785.1
VIT 200s0264g00015.1
AT5G38990.1
AT5G39000.1
Aco001484.1
Aco014392.1
AT5G54380.1
CIL1078S0101
VIT 219s0014g03400.1
CIL0954S0001
CIL0951S0111
CIL1207S0059
VIT 207s0129g00960.1
VIT 207s0129g00950.1
VIT 207s0129g00890.1
VIT 207s0129g00920.1
VIT 207s0129g00910.1
VIT 207s0129g00870.1
VIT 207s0129g00880.1
VIT 207s0129g00990.1
VIT 207s0129g00970.1
VIT 200s1542g00010.1
VIT 200s1542g00020.1
AT2G23200.1
CIL0990S0002
Aco016615.1
AT5G24010.1
VIT 216s0050g01020.1
AT3G46290.1
AT5G59700.1
VIT 206s0004g03920.1
CIL1050S0021
VIT 213s0067g03780.1
CIL1546S0029
AT2G39360.1
Aco010993.1
AT1G30570.1
CIL1286S0036
VIT 210s0003g04990.1
Aco005072.1
Aco014821.1
Aco008525.1
VIT 217s0000g06620.1
AT5G61350.1
AT2G21480.1
AT4G39110.1
Aco018550.1
CIL0917S0053
VIT 203s0038g04340.1
AT3G04690.1
AT5G28680.1
CIL0935S0072
CIL1009S0054
VIT 205s0077g00970.1
VIT 207s0005g01640.1
Aco002557.1
Aco018669.1
VIT 208s0040g00010.1
CIL1324S0036
AT3G51550.1
CIL0909S0241
CIL0909S0242
VIT 214s0068g00010.1
VIT 214s0083g01195.1
VIT 214s0068g00090.1
CIL1201S0040
CIL0895S0004
CIL1321S0046
CIL0568S0001
CIL1101S0002
VIT 214s0068g00110.1
VIT 214s0068g00130.1
VIT 214s0083g01220.1
VIT 201s0244g00090.1
VIT 214s0068g00030.1
VIT 214s0083g01210.1
VIT 201s0011g00280.1
AT4G29180.2
AT4G29450.1
VIT 209s0002g02840.1
VIT 209s0002g02900.1
CIL1131S0100
CIL0004S0023
CIL0697S0001
CIL1131S0101
VIT 209s0002g02710.1
VIT 209s0002g02730.1
AT1G49100.1
AT1G51810.1
AT1G51830.1
AT1G51820.1
AT1G51805.1
AT1G51850.1
AT2G04300.1
AT3G21340.1
AT4G20450.1
AT2G28960.1
AT2G29000.1
AT1G07560.1
AT5G16900.1
AT5G59660.1
AT5G59670.1
AT5G59680.1
AT5G59650.1
AT3G45920.1
AT3G46330.1
AT3G46350.1
AT3G46340.1
AT3G46370.1
AT3G46400.1
AT3G46410.1
AT3G46420.1
AT1G07550.1
AT2G14440.1
AT2G14510.1
AT2G28970.1
AT2G28990.1
AT1G51910.1
AT1G51890.1
AT1G51880.1
AT1G51860.1
AT1G51870.1
AT1G51800.1
AT1G05700.1
AT2G19190.1
AT4G29990.1
AT2G19210.1
AT2G19230.1
AT1G51620.2
AT1G51790.1

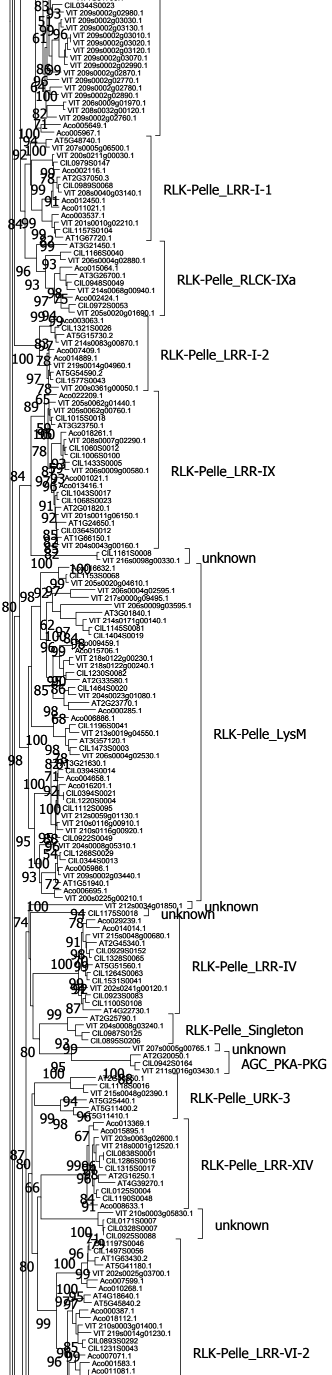
RLK-Pelle_LRR-I-1
RLK-Pelle_RLCK-IXa
RLK-Pelle_LRR-I-2
RLK-Pelle_LRR-IX
unknown
RLK-Pelle_LysM
unknown
unknown
RLK-Pelle_LRR-IV
RLK-Pelle_Singleton
unknown
AGC_PKA-PKG
RLK-Pelle_URK-3
RLK-Pelle_LRR-XIV
unknown
RLK-Pelle_LRR-VI-2

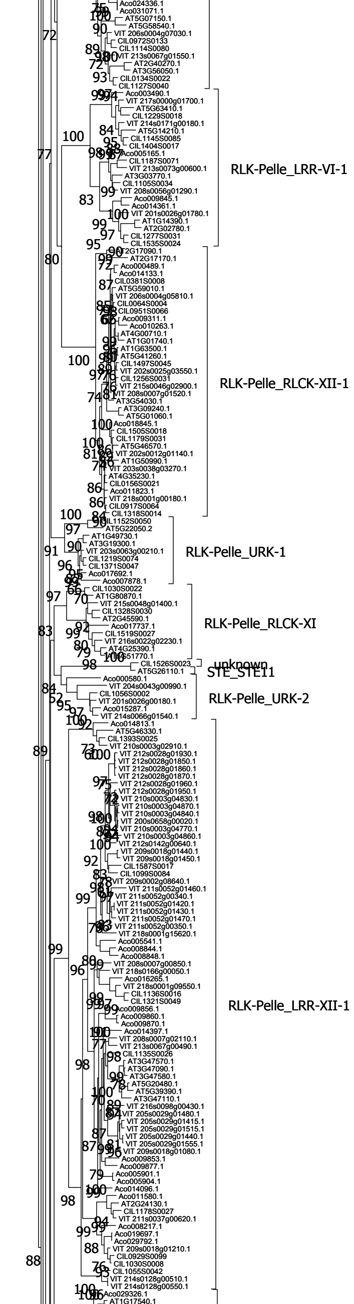

RLK-Pelle_LRR-VI-1
RLK-Pelle_RLCK-XII-1
RLK-Pelle_URK-1
RLK-Pelle_RLCK-XI
unknown
STE_STE11
RLK-Pelle_URK-2
RLK-Pelle_LRR-XII-1
Aco024336.1
Aco031071.1
AT5G07150.1
AT5G58540.1
VIT 206s0004g07030.1
CIL0972S0133
CIL1114S0080
VIT 213s0067g01550.1
AT2G40270.1
AT3G56050.1
CIL0134S0022
CIL1127S0040
Aco003490.1
VIT 217s0000g01700.1
AT5G63410.1
CIL1229S0018
VIT 214s0171g00180.1
AT5G14210.1
CIL1145S0085
CIL1404S0017
Aco005165.1
CIL1187S0071
VIT 213s0073g00600.1
AT3G03770.1
CIL1105S0034
VIT 208s0056g01290.1
Aco009845.1
Aco014361.1
VIT 201s0026g01780.1
AT1G14390.1
AT2G02780.1
CIL1277S0031
CIL1535S0024
AT2G17090.1
AT2G17170.1
Aco000489.1
Aco014133.1
CIL0381S0008
AT5G59010.1
VIT 206s0004g05810.1
CIL0064S0004
CIL0951S0066
Aco009311.1
Aco010263.1
AT4G00710.1
AT1G01740.1
AT1G63500.1
AT5G41260.1
CIL1497S0045
VIT 202s0025g03550.1
CIL1256S0031
VIT 215s0046g02900.1
VIT 208s0007g01520.1
AT3G54030.1
AT3G09240.1
AT5G01060.1
Aco018845.1
CIL1505S0018
CIL1179S0031
AT5G46570.1
VIT 202s0012g01140.1
AT1G50990.1
VIT 203s0038g03270.1
AT4G35230.1
CIL0156S0021
Aco011823.1
VIT 218s0001g00180.1
CIL0917S0064
CIL1318S0014
CIL1152S0050
AT5G22050.2
AT1G49730.1
AT3G19300.1
VIT 203s0063g00210.1
CIL1219S0074
CIL1371S0047
Aco017692.1
Aco007878.1
CIL1030S0022
AT1G80870.1
VIT 215s0048g01400.1
CIL1328S0030
AT2G45590.1
Aco017737.1
CIL1519S0027
VIT 216s0022g02230.1
AT4G25390.1
AT5G51770.1
CIL1526S0023
AT5G26110.1
Aco000580.1
VIT 204s0043g00990.1
CIL1056S0002
VIT 201s0026g00180.1
Aco015287.1
VIT 214s0066g01540.1
Aco014813.1
AT5G46330.1
CIL1393S0025
VIT 210s0003g02910.1
VIT 212s0028g01930.1
VIT 212s0028g01850.1
VIT 212s0028g01860.1
VIT 212s0028g01870.1
VIT 212s0028g01960.1
VIT 212s0028g01950.1
VIT 210s0003g04830.1
VIT 210s0003g04870.1
VIT 210s0003g04840.1
VIT 200s0658g00020.1
VIT 210s0003g04770.1
VIT 210s0003g04860.1
VIT 212s0142g00640.1
VIT 209s0018g01440.1
VIT 209s0018g01450.1
CIL1587S0017
CIL1099S0084
VIT 209s0002g08640.1
VIT 211s0052g01460.1
VIT 211s0052g00340.1
VIT 211s0052g01420.1
VIT 211s0052g01430.1
VIT 211s0052g01470.1
VIT 211s0052g00350.1
VIT 218s0001g15620.1
Aco005541.1
Aco008844.1
Aco008848.1
VIT 208s0007g00850.1
VIT 218s0166g00050.1
Aco016265.1
VIT 218s0001g09550.1
CIL1136S0016
CIL1321S0049
Aco009856.1
Aco009860.1
Aco009870.1
Aco014397.1
VIT 208s0007g02110.1
VIT 213s0067g00490.1
CIL1135S0026
AT3G47570.1
AT3G47090.1
AT3G47580.1
AT5G20480.1
AT5G39390.1
AT3G47110.1
VIT 216s0098g00430.1
VIT 205s0029g01480.1
VIT 205s0029g01415.1
VIT 205s0029g01515.1
VIT 205s0029g01440.1
VIT 205s0029g01555.1
VIT 209s0018g01080.1
Aco009853.1
Aco009877.1
Aco005901.1
Aco005904.1
Aco014096.1
Aco011580.1
AT2G24130.1
CIL1178S0027
VIT 211s0037g00620.1
Aco008217.1
Aco019697.1
Aco029792.1
VIT 209s0018g01210.1
CIL0929S0099
CIL1030S0008
CIL1055S0042
VIT 214s0128g00510.1
VIT 214s0128g00550.1
Aco029326.1
AT1G17540.1

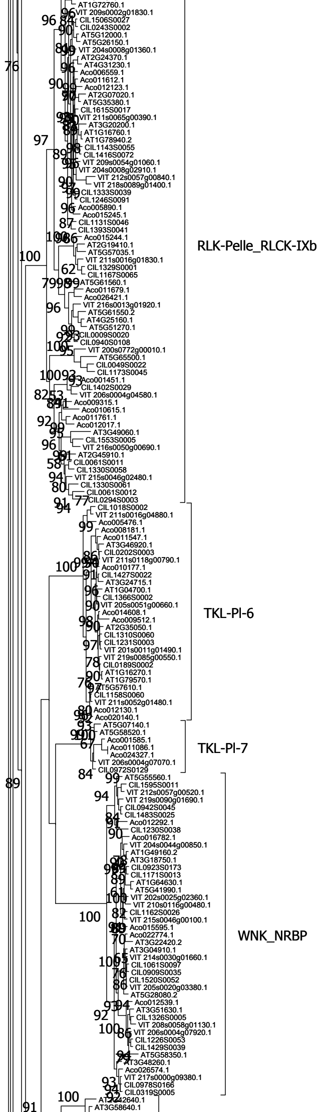
RLK-Pelle_RLCK-IXb
TKL-Pl-6
TKL-Pl-7
WNK_NRBP

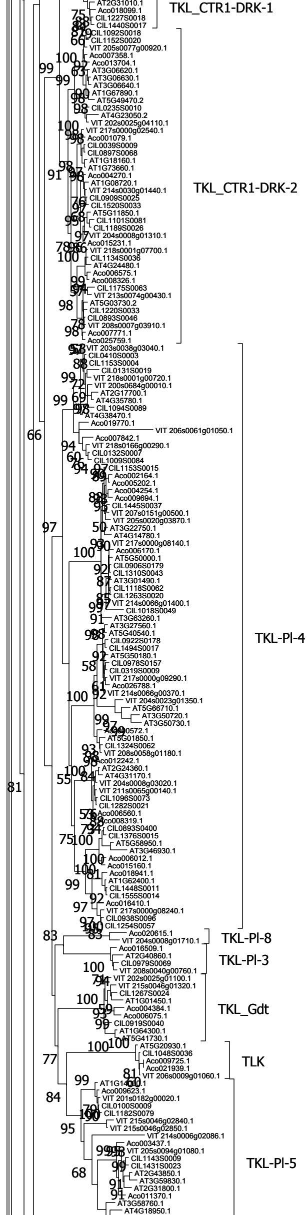

TKL_CTR1-DRK-1
TKL_CTR1-DRK-2
TKL-Pl-4
TKL-Pl-8
TKL-Pl-3
TKL_Gdt
TLK
TKL-Pl-5

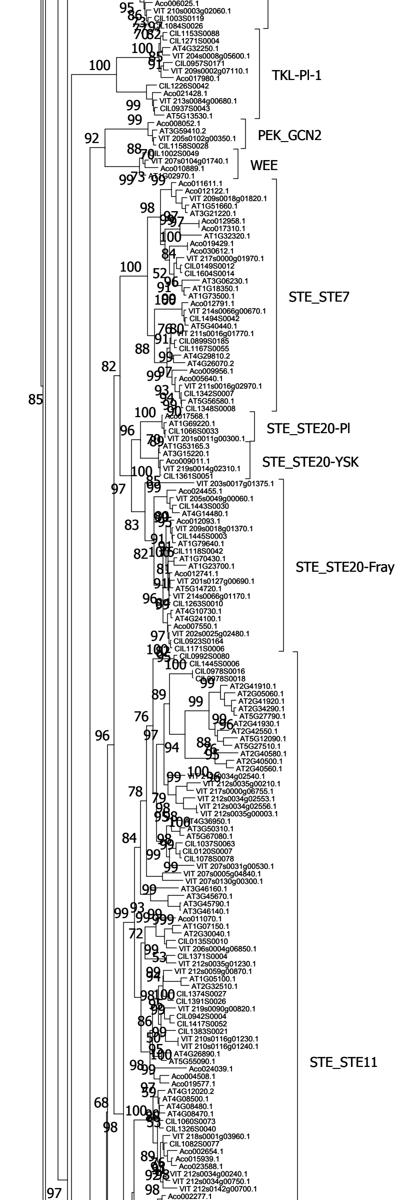
TKL-Pl-1
PEK_GCN2
WEE
STE_STE7
STE_STE20-Pl
STE_STE20-YSK
STE_STE20-Fray
STE_STE11

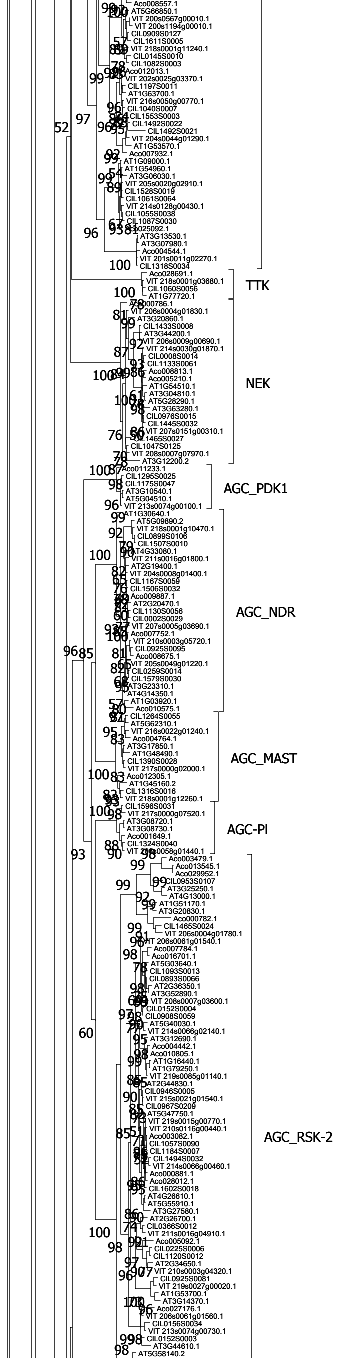
Aco008557.1
AT5G66850.1
VIT 200s0567g00010.1
VIT 200s1194g00010.1
CIL0909S0127
CIL1611S0005
VIT 218s0001g11240.1
CIL0145S0010
CIL1082S0003
Aco012013.1
VIT 202s0025g03370.1
CIL1197S0011
AT1G63700.1
VIT 216s0050g00770.1
CIL1040S0007
CIL1553S0003
CIL1492S0022
CIL1492S0021
VIT 204s0044g01290.1
AT1G53570.1
Aco007932.1
AT1G09000.1
AT1G54960.1
AT3G06030.1
VIT 205s0020g02910.1
CIL1528S0019
CIL1061S0064
VIT 214s0128g00430.1
CIL1055S0038
CIL1087S0030
AT3G13530.1
AT3G07980.1
Aco004544.1
VIT 201s0011g02270.1
CIL1318S0034
Aco028691.1
VIT 218s0001g03680.1
CIL1060S0056
AT1G77720.1
TTK
VIT 206s0004g01830.1
AT3G20860.1
CIL1433S0008
AT3G44200.1
VIT 206s0009g00690.1
VIT 214s0030g01870.1
CIL0008S0014
CIL1133S0061
Aco008813.1
Aco005210.1
AT1G54510.1
AT3G04810.1
AT5G28290.1
AT3G63280.1
CIL0976S0015
CIL1445S0032
VIT 207s0151g00310.1
CIL1465S0027
CIL1047S0125
VIT 208s0007g07970.1
AT3G12200.2
NEK
Aco011233.1
CIL1295S0025
CIL1175S0047
AT3G10540.1
AT5G04510.1
VIT 213s0074g00100.1
AGC_PDK1
AT1G30640.1
AT5G09890.2
VIT 218s0001g10470.1
CIL0899S0106
CIL1507S0010
AT4G33080.1
VIT 211s0016g01800.1
AT2G19400.1
VIT 204s0008g01400.1
CIL1167S0059
CIL1506S0032
Aco009887.1
AT2G20470.1
CIL1130S0056
CIL0002S0029
VIT 207s0005g03690.1
Aco007752.1
VIT 210s0003g05720.1
CIL0925S0095
Aco008675.1
VIT 205s0049g01220.1
CIL0259S0014
CIL1579S0030
AT3G23310.1
AT4G14350.1
AGC_NDR
AT1G03920.1
Aco010575.1
CIL1264S0055
AT5G62310.1
VIT 216s0022g01240.1
Aco004764.1
AT3G17850.1
AT1G48490.1
CIL1390S0028
VIT 217s0000g02000.1
Aco012305.1
AT1G45160.2
CIL1316S0016
VIT 218s0001g12260.1
AGC_MAST
CIL1596S0031
VIT 217s0000g07520.1
AT3G08720.1
AT3G08730.1
Aco001649.1
CIL1324S0040
AGC-PI
Aco003479.1
Aco013545.1
Aco029952.1
CIL0953S0107
AT3G25250.1
AT4G13000.1
AT1G51170.1
AT3G20830.1
Aco000782.1
CIL1465S0024
VIT 206s0004g01780.1
VIT 206s0061g01540.1
Aco007784.1
Aco016701.1
AT5G03640.1
CIL1093S0013
CIL0893S0066
AT2G36350.1
AT3G52890.1
VIT 208s0007g03600.1
CIL0152S0004
CIL0908S0059
AT5G40030.1
VIT 214s0066g02140.1
AT3G12690.1
Aco004442.1
Aco010805.1
AT1G16440.1
AT1G79250.1
VIT 219s0085g01140.1
AT2G44830.1
CIL0946S0005
VIT 215s0021g01540.1
CIL0967S0209
AT5G47750.1
VIT 219s0015g00770.1
VIT 210s0116g00440.1
Aco003082.1
CIL1057S0090
CIL1184S0007
CIL1494S0032
VIT 214s0066g00460.1
Aco000881.1
Aco028012.1
CIL1602S0018
AT4G26610.1
AT5G55910.1
AT3G27580.1
AT2G26700.1
CIL0366S0012
VIT 211s0016g04910.1
Aco005092.1
CIL0225S0006
CIL1120S0012
AT2G34650.1
VIT 210s0003g04320.1
CIL0925S0081
VIT 219s0027g00020.1
AT1G53700.1
AT3G14370.1
Aco027176.1
VIT 206s0061g01560.1
CIL0156S0034
VIT 213s0074g00730.1
CIL0152S0003
AT3G44610.1
AT5G58140.2
AGC_RSK-2

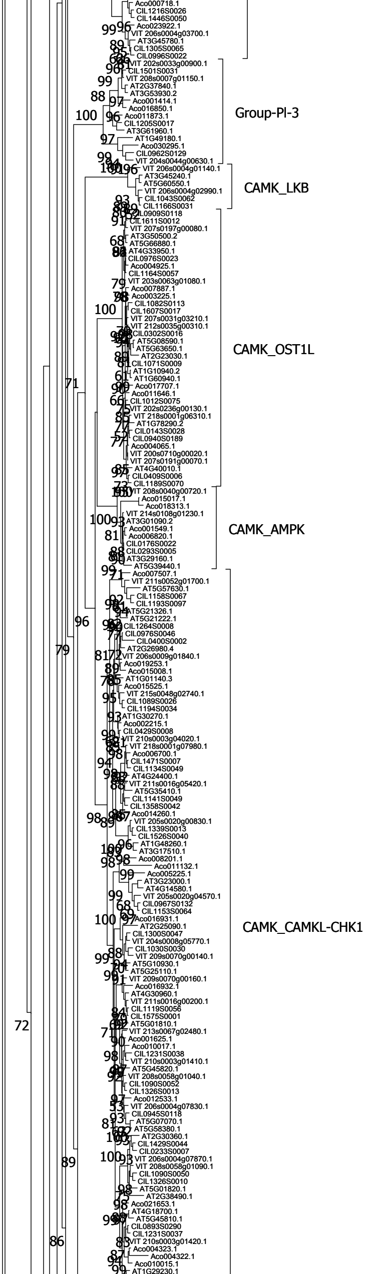

Aco000718.1
CIL1216S0026
CIL1446S0050
Aco023922.1
VIT 206s0004g03700.1
AT3G45780.1
CIL1305S0065
CIL0996S0022
VIT 202s0033g00900.1
CIL1501S0031
VIT 208s0007g01150.1
AT2G37840.1
AT3G53930.2
Aco001414.1
Aco016850.1
Aco011873.1
CIL1205S0017
AT3G61960.1
AT1G49180.1
Aco030295.1
CIL0962S0129
VIT 204s0044g00630.1
Group-Pl-3
VIT 206s0004g01140.1
AT3G45240.1
AT5G60550.1
VIT 206s0004g02990.1
CIL1043S0062
CIL1166S0031
CAMK_LKB
CIL0909S0118
CIL1611S0012
VIT 207s0197g00080.1
AT3G50500.2
AT5G66880.1
AT4G33950.1
CIL0976S0023
Aco004925.1
CIL1164S0057
VIT 203s0063g01080.1
Aco007887.1
Aco003225.1
CIL1082S0113
CIL1607S0017
VIT 207s0031g03210.1
VIT 212s0035g00310.1
CIL0302S0016
AT5G08590.1
AT5G63650.1
AT2G23030.1
CIL1071S0009
AT1G10940.2
AT1G60940.1
Aco017707.1
Aco011646.1
CIL1012S0075
VIT 202s0236g00130.1
VIT 218s0001g06310.1
AT1G78290.2
CIL0143S0028
CIL0940S0189
Aco004065.1
VIT 200s0710g00020.1
VIT 207s0191g00070.1
AT4G40010.1
CIL0409S0006
CIL1189S0070
CAMK_OST1L
VIT 208s0040g00720.1
Aco015017.1
Aco018313.1
VIT 214s0108g01230.1
AT3G01090.2
Aco001549.1
Aco006820.1
CIL0176S0022
CIL0293S0005
AT3G29160.1
AT5G39440.1
CAMK_AMPK
Aco007507.1
VIT 211s0052g01700.1
AT5G57630.1
CIL1158S0067
CIL1193S0097
AT5G21326.1
AT5G21222.1
CIL1264S0008
CIL0976S0046
CIL0400S0002
AT2G26980.4
VIT 206s0009g01840.1
Aco019253.1
Aco015008.1
AT1G01140.3
Aco015525.1
VIT 215s0048g02740.1
CIL1089S0026
CIL1194S0034
AT1G30270.1
Aco002215.1
CIL0429S0008
VIT 210s0003g04020.1
VIT 218s0001g07980.1
Aco006700.1
CIL1471S0007
CIL1134S0049
AT4G24400.1
VIT 211s0016g05420.1
AT5G35410.1
CIL1141S0049
CIL1358S0042
Aco014260.1
VIT 205s0020g00830.1
CIL1339S0013
CIL1526S0040
AT1G48260.1
AT3G17510.1
Aco008201.1
Aco011132.1
Aco005225.1
AT3G23000.1
AT4G14580.1
VIT 205s0020g04570.1
CIL0967S0132
CIL1153S0064
Aco016931.1
AT2G25090.1
CIL1300S0047
VIT 204s0008g05770.1
CIL1030S0030
VIT 209s0070g00140.1
AT5G10930.1
AT5G25110.1
VIT 209s0070g00160.1
Aco016932.1
AT4G30960.1
VIT 211s0016g00200.1
CIL1119S0056
CIL1575S0001
AT5G01810.1
VIT 213s0067g02480.1
Aco001625.1
Aco010017.1
CIL1231S0038
VIT 210s0003g01410.1
AT5G45820.1
VIT 208s0058g01040.1
CIL1090S0052
CIL1326S0013
Aco012533.1
VIT 206s0004g07830.1
CIL0945S0118
AT5G07070.1
AT5G58380.1
AT2G30360.1
CIL1429S0044
CIL0233S0007
VIT 206s0004g07870.1
VIT 208s0058g01090.1
CIL1090S0050
CIL1326S0010
AT5G01820.1
AT2G38490.1
Aco021653.1
AT4G18700.1
AT5G45810.1
CIL0893S0290
CIL1231S0037
VIT 210s0003g01420.1
Aco004323.1
Aco004322.1
Aco010015.1
AT1G29230.1
CAMK_CAMKL-CHK1

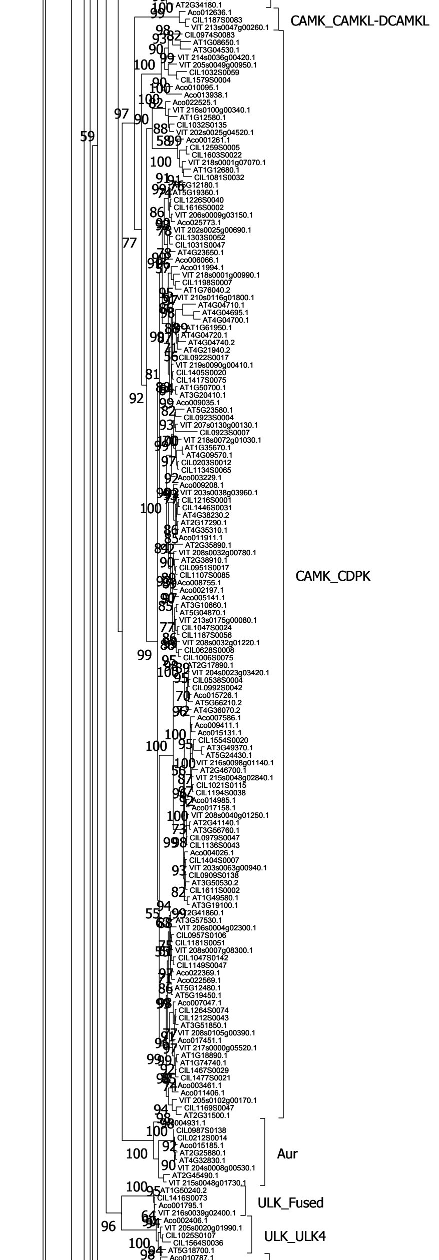
CAMK_CAMKL-DCAMKL
CAMK_CDPK
Aur
ULK_Fused
ULK_ULK4

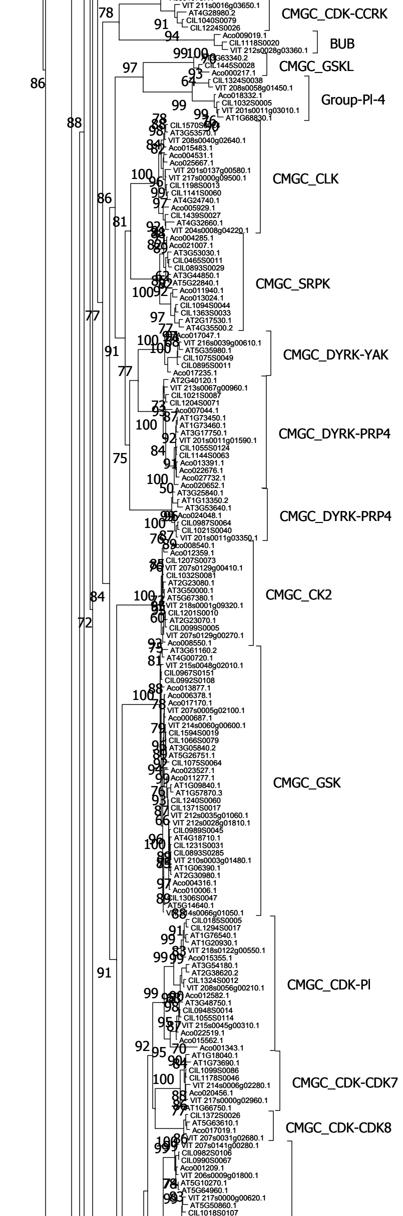
VIT 211s0016g03650.1
AT4G28980.2
CIL1040S0079
CIL1224S0026
CMGC_CDK-CCRK
Aco009019.1
CIL1118S0020
VIT 212s0028g03360.1
BUB
CIL1445S0028
Aco000217.1
CMGC_GSKL
CIL1324S0038
VIT 208s0058g01450.1
Aco018332.1
CIL1032S0005
VIT 201s0011g03010.1
AT1G68830.1
Group-Pl-4
AT3G53570.1
VIT 208s0040g02640.1
Aco015483.1
Aco004531.1
Aco025667.1
VIT 201s0137g00580.1
VIT 217s0000g09500.1
CIL1198S0013
CIL1141S0060
AT4G24740.1
Aco005929.1
CIL1439S0027
AT4G32660.1
VIT 204s0008g04220.1
CMGC_CLK
Aco004285.1
Aco021007.1
AT3G53030.1
CIL0465S0011
CIL0893S0029
AT3G44850.1
AT5G22840.1
Aco011940.1
Aco013024.1
CIL1094S0044
CIL1363S0033
AT2G17530.1
AT4G35500.2
CMGC_SRPK
VIT 216s0039g00610.1
AT5G35980.1
CIL1075S0049
CIL0895S0011
Aco017235.1
CMGC_DYRK-YAK
AT2G40120.1
VIT 213s0067g00960.1
CIL1021S0087
CIL1204S0071
Aco007044.1
AT1G73450.1
AT1G73460.1
AT3G17750.1
VIT 201s0011g01590.1
CIL1055S0124
CIL1144S0063
Aco013391.1
Aco022676.1
Aco027732.1
Aco020652.1
CMGC_DYRK-PRP4
AT3G25840.1
AT1G13350.2
AT3G53640.1
CIL0987S0064
CIL1021S0040
VIT 201s0011g03350.1
CMGC_DYRK-PRP4
Aco012359.1
CIL1207S0073
VIT 207s0129g00410.1
CIL1032S0081
AT2G23080.1
AT3G50000.1
AT5G67380.1
VIT 218s0001g09320.1
CIL1201S0010
AT2G23070.1
CIL0099S0005
VIT 207s0129g00270.1
Aco008550.1
CMGC_CK2
AT3G61160.2
AT4G00720.1
VIT 215s0048g02010.1
CIL0967S0151
CIL0992S0108
Aco013877.1
Aco006378.1
Aco017170.1
VIT 207s0005g02100.1
Aco000687.1
VIT 214s0060g00600.1
CIL1594S0019
CIL1066S0079
AT3G05840.2
AT5G26751.1
CIL1075S0064
Aco023527.1
Aco011277.1
AT1G09840.1
AT1G57870.3
CIL1240S0060
CIL1371S0017
VIT 212s0035g01060.1
VIT 212s0028g01810.1
CIL0989S0045
AT4G18710.1
CIL1231S0031
CIL0893S0285
VIT 210s0003g01480.1
AT1G06390.1
AT2G30980.1
Aco004316.1
Aco010006.1
CIL1306S0047
AT5G14640.1
CMGC_GSK
CIL0185S0005
CIL1294S0017
AT1G76540.1
AT1G20930.1
VIT 218s0122g00550.1
Aco015355.1
AT3G54180.1
AT2G38620.2
CIL1324S0012
VIT 208s0056g00210.1
Aco012582.1
AT3G48750.1
CIL0948S0014
CIL1055S0114
VIT 215s0045g00310.1
Aco022519.1
Aco015562.1
Aco001343.1
CMGC_CDK-Pl
AT1G18040.1
AT1G73690.1
CIL1099S0086
CIL1178S0046
VIT 214s0006g02280.1
Aco020456.1
VIT 217s0000g02960.1
AT1G66750.1
CMGC_CDK-CDK7
CIL1372S0026
AT5G63610.1
Aco017019.1
VIT 207s0031g02680.1
CMGC_CDK-CDK8
VIT 207s0141g00280.1
CIL0982S0106
CIL0990S0067
Aco001209.1
VIT 206s0009g01800.1
AT5G10270.1
AT5G64960.1
VIT 217s0000g00620.1
AT5G50860.1
CIL1018S0107

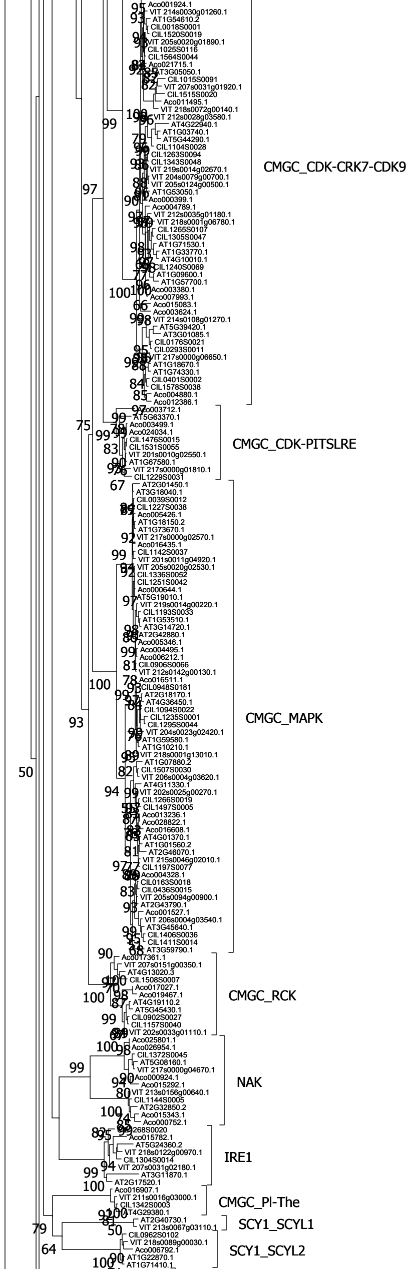
Aco001924.1
VIT 214s0030g01260.1
AT1G54610.2
CIL0018S0001
CIL1520S0019
VIT 205s0020g01890.1
CIL1025S0116
CIL1564S0044
Aco021715.1
AT3G05050.1
CIL1015S0091
VIT 207s0031g01920.1
CIL1515S0020
Aco011495.1
VIT 218s0072g00140.1
VIT 212s0028g03580.1
AT4G22940.1
AT1G03740.1
AT5G44290.1
CIL1104S0028
CIL1263S0094
CIL1343S0048
VIT 219s0014g02670.1
VIT 204s0079g00700.1
VIT 205s0124g00500.1
AT1G53050.1
Aco000399.1
Aco004789.1
VIT 212s0035g01180.1
VIT 218s0001g06780.1
CIL1265S0107
CIL1305S0047
AT1G71530.1
AT1G33770.1
AT4G10010.1
CIL1240S0069
AT1G09600.1
AT1G57700.1
Aco003380.1
Aco007993.1
Aco015083.1
Aco003624.1
VIT 214s0108g01270.1
AT5G39420.1
AT3G01085.1
CIL0176S0021
CIL0293S0011
VIT 217s0000g06650.1
AT1G18670.1
AT1G74330.1
CIL0401S0002
CIL1578S0038
Aco004880.1
Aco012386.1
CMGC_CDK-CRK7-CDK9
Aco003712.1
AT5G63370.1
Aco003499.1
Aco024034.1
CIL1476S0015
CIL1531S0055
VIT 201s0010g02550.1
AT1G67580.1
VIT 217s0000g01810.1
CIL1229S0031
CMGC_CDK-PITSLRE
AT2G01450.1
AT3G18040.1
CIL0039S0012
CIL1227S0038
Aco005426.1
AT1G18150.2
AT1G73670.1
VIT 217s0000g02570.1
Aco016435.1
CIL1142S0037
VIT 201s0011g04920.1
VIT 205s0020g02530.1
CIL1336S0052
CIL1251S0042
Aco000644.1
AT5G19010.1
VIT 219s0014g00220.1
CIL1193S0033
AT1G53510.1
AT3G14720.1
AT2G42880.1
Aco005346.1
Aco004495.1
Aco006212.1
CIL0906S0066
VIT 212s0142g00130.1
Aco016511.1
CIL0948S0181
AT2G18170.1
AT4G36450.1
CIL1094S0022
CIL1235S0001
CIL1295S0044
VIT 204s0023g02420.1
AT1G59580.1
AT1G10210.1
VIT 218s0001g13010.1
AT1G07880.2
CIL1507S0030
VIT 206s0004g03620.1
AT4G11330.1
VIT 202s0025g00270.1
CIL1266S0019
CIL1497S0005
Aco013236.1
Aco028822.1
Aco016608.1
AT4G01370.1
AT1G01560.2
AT2G46070.1
VIT 215s0046g02010.1
CIL1197S0077
Aco004328.1
CIL0163S0018
CIL0436S0015
VIT 205s0094g00900.1
AT2G43790.1
Aco001527.1
VIT 206s0004g03540.1
AT3G45640.1
CIL1406S0036
CIL1411S0014
AT3G59790.1
CMGC_MAPK
Aco017361.1
VIT 207s0151g00350.1
AT4G13020.3
CIL1508S0007
Aco017027.1
Aco019467.1
AT4G19110.2
AT5G45430.1
CIL0902S0027
CIL1157S0040
VIT 202s0033g01110.1
CMGC_RCK
Aco025801.1
Aco026954.1
CIL1372S0045
AT5G08160.1
VIT 217s0000g04670.1
Aco000924.1
Aco015292.1
VIT 213s0156g00640.1
CIL1144S0005
AT2G32850.2
Aco015343.1
Aco000752.1
NAK
Aco015782.1
AT5G24360.2
VIT 218s0122g00970.1
CIL1304S0014
VIT 207s0031g02180.1
AT3G11870.1
AT2G17520.1
IRE1
Aco016907.1
VIT 211s0016g03000.1
CIL1342S0003
AT4G29380.1
CMGC_Pl-The
AT2G40730.1
VIT 213s0067g03110.1
SCY1_SCYL1
CIL0962S0102
VIT 218s0089g00030.1
Aco006792.1
AT1G22870.1
AT1G71410.1
SCY1_SCYL2

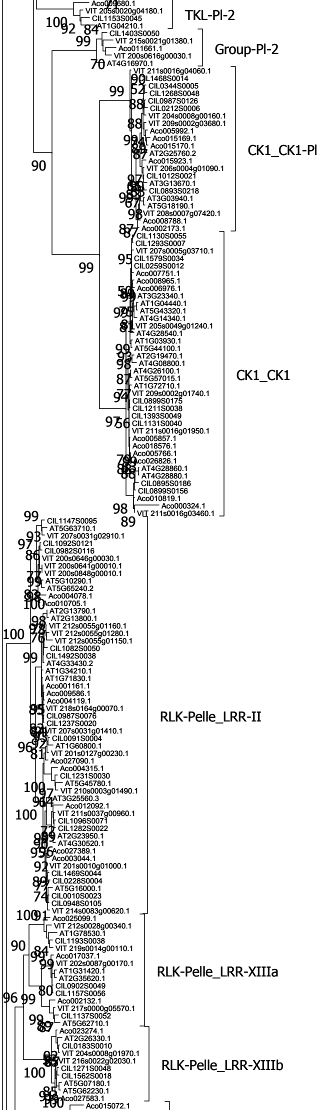

TKL-Pl-2
Group-Pl-2
CK1_CK1-Pl
CK1_CK1
RLK-Pelle_LRR-II
RLK-Pelle_LRR-XIIIa
RLK-Pelle_LRR-XIIIb
VIT 205s0020g04180.1
CIL1153S0045
AT1G04210.1
CIL1403S0050
VIT 215s0021g01380.1
Aco011661.1
VIT 200s0616g00030.1
AT4G16970.1
VIT 211s0016g04060.1
CIL0344S0005
CIL1268S0048
CIL0987S0126
CIL0212S0006
VIT 204s0008g00160.1
VIT 209s0002g03680.1
Aco005992.1
Aco015169.1
Aco015170.1
AT2G25760.2
Aco015923.1
VIT 206s0004g01090.1
CIL1012S0021
AT3G13670.1
CIL0893S0218
AT3G03940.1
AT5G18190.1
VIT 208s0007g07420.1
Aco008788.1
Aco002173.1
CIL1130S0055
CIL1293S0007
VIT 207s0005g03710.1
CIL1579S0034
CIL0259S0012
Aco007751.1
Aco008965.1
Aco006976.1
AT3G23340.1
AT1G04440.1
AT5G43320.1
AT4G14340.1
VIT 205s0049g01240.1
AT4G28540.1
AT1G03930.1
AT5G44100.1
AT2G19470.1
AT4G08800.1
AT4G26100.1
AT5G57015.1
AT1G72710.1
VIT 209s0002g01740.1
CIL0899S0175
CIL1211S0038
CIL1393S0049
CIL1131S0040
VIT 211s0016g01950.1
Aco005857.1
Aco018576.1
Aco005766.1
Aco026826.1
AT4G28860.1
AT4G28880.1
CIL0895S0186
CIL0899S0156
Aco010819.1
Aco000324.1
VIT 211s0016g03460.1
CIL1147S0095
AT5G63710.1
VIT 207s0031g02910.1
CIL1092S0121
CIL0982S0116
VIT 200s0646g00030.1
VIT 200s0641g00010.1
VIT 200s0848g00010.1
AT5G10290.1
AT5G65240.2
Aco004078.1
Aco010705.1
AT2G13790.1
AT2G13800.1
VIT 212s0055g01160.1
VIT 212s0055g01280.1
VIT 212s0055g01150.1
CIL1082S0050
CIL1492S0038
AT4G33430.2
AT1G34210.1
AT1G71830.1
Aco001161.1
Aco009586.1
Aco004119.1
VIT 218s0164g00070.1
CIL0987S0076
CIL1237S0020
VIT 207s0031g01410.1
CIL0091S0004
AT1G60800.1
VIT 201s0127g00230.1
Aco027090.1
Aco004315.1
CIL1231S0030
AT5G45780.1
VIT 210s0003g01490.1
AT3G25560.3
Aco012092.1
VIT 211s0037g00960.1
CIL1096S0071
CIL1282S0022
AT2G23950.1
AT4G30520.1
Aco027389.1
Aco003044.1
VIT 201s0010g01000.1
CIL1469S0044
CIL0228S0004
AT5G16000.1
CIL0010S0023
CIL0948S0105
VIT 214s0083g00620.1
Aco025099.1
VIT 212s0028g00340.1
AT1G78530.1
CIL1193S0038
VIT 219s0014g00110.1
Aco017037.1
VIT 202s0087g00170.1
AT1G31420.1
AT2G35620.1
CIL0902S0049
CIL1157S0056
Aco002132.1
VIT 217s0000g05570.1
CIL1137S0052
AT5G62710.1
Aco023274.1
AT2G26330.1
CIL0183S0010
VIT 204s0008g01970.1
VIT 216s0022g02030.1
CIL1271S0048
CIL1562S0018
AT5G07180.1
AT5G62230.1
Aco027583.1
Aco015072.1

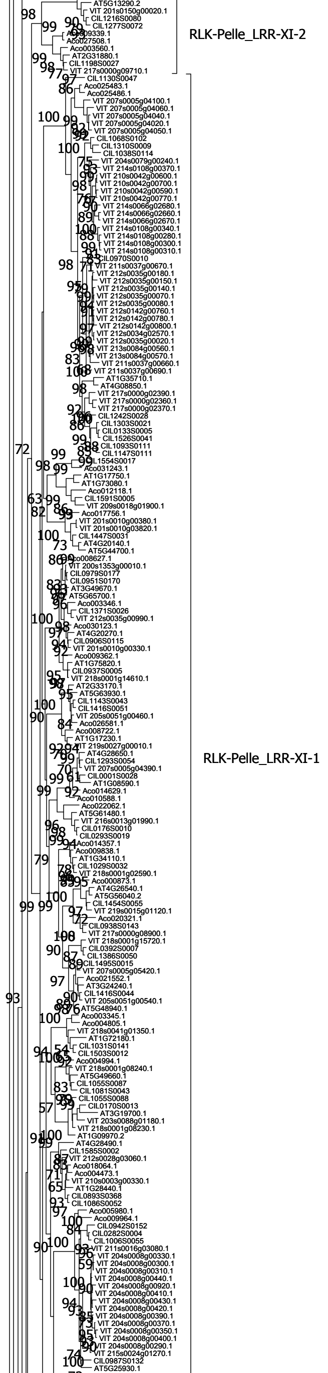

RLK-Pelle_LRR-XI-2
RLK-Pelle_LRR-XI-1
AT5G13290.2
VIT 201s0150g00020.1
CIL1216S0080
CIL1277S0072
Aco009339.1
Aco027508.1
Aco003560.1
AT2G31880.1
CIL1198S0027
VIT 217s0000g09710.1
CIL1130S0047
Aco025483.1
Aco025486.1
VIT 207s0005g04100.1
VIT 207s0005g04060.1
VIT 207s0005g04040.1
VIT 207s0005g04020.1
VIT 207s0005g04050.1
CIL1068S0102
CIL1310S0009
CIL1038S0114
VIT 204s0079g00240.1
VIT 214s0108g00370.1
VIT 210s0042g00600.1
VIT 210s0042g00700.1
VIT 210s0042g00590.1
VIT 210s0042g00770.1
VIT 214s0066g02680.1
VIT 214s0066g02660.1
VIT 214s0066g02670.1
VIT 214s0108g00340.1
VIT 214s0108g00280.1
VIT 214s0108g00300.1
VIT 214s0108g00310.1
CIL0970S0010
VIT 211s0037g00670.1
VIT 212s0035g00180.1
VIT 212s0035g00150.1
VIT 212s0035g00140.1
VIT 212s0035g00070.1
VIT 212s0035g00080.1
VIT 212s0142g00760.1
VIT 212s0142g00780.1
VIT 212s0142g00800.1
VIT 212s0034g02570.1
VIT 212s0035g00020.1
VIT 213s0084g00560.1
VIT 213s0084g00570.1
VIT 211s0037g00660.1
VIT 211s0037g00690.1
AT1G35710.1
AT4G08850.1
VIT 217s0000g02390.1
VIT 217s0000g02360.1
VIT 217s0000g02370.1
CIL1242S0028
CIL1303S0021
CIL0133S0005
CIL1526S0041
CIL1093S0111
CIL1147S0111
CIL1554S0017
Aco031243.1
AT1G17750.1
AT1G73080.1
Aco012118.1
CIL1591S0005
VIT 209s0018g01900.1
Aco017756.1
VIT 201s0010g00380.1
VIT 201s0010g03820.1
CIL1447S0031
AT4G20140.1
AT5G44700.1
Aco008627.1
VIT 200s1353g00010.1
CIL0979S0177
CIL0951S0170
AT3G49670.1
AT5G65700.1
Aco003346.1
CIL1371S0026
VIT 212s0035g00990.1
Aco030123.1
AT4G20270.1
CIL0906S0115
VIT 201s0010g00330.1
Aco009362.1
AT1G75820.1
CIL0937S0005
VIT 218s0001g14610.1
AT2G33170.1
AT5G63930.1
CIL1143S0043
CIL1416S0051
VIT 205s0051g00460.1
Aco026581.1
Aco008722.1
AT1G17230.1
VIT 219s0027g00010.1
AT4G28650.1
CIL1293S0054
VIT 207s0005g04390.1
CIL0001S0028
AT1G08590.1
Aco014629.1
Aco010588.1
Aco022062.1
AT5G61480.1
VIT 216s0013g01990.1
CIL0176S0010
CIL0293S0019
Aco014357.1
Aco009838.1
AT1G34110.1
CIL1029S0032
VIT 218s0001g02590.1
Aco000873.1
AT4G26540.1
AT5G56040.2
CIL1454S0055
VIT 219s0015g01120.1
Aco020321.1
CIL0938S0143
VIT 217s0000g08900.1
VIT 218s0001g15720.1
CIL0392S0007
CIL1386S0050
CIL1495S0015
VIT 207s0005g05420.1
Aco021552.1
AT3G24240.1
CIL1416S0044
VIT 205s0051g00540.1
AT5G48940.1
Aco003345.1
Aco004805.1
VIT 218s0041g01350.1
AT1G72180.1
CIL1031S0141
CIL1503S0012
Aco004994.1
VIT 218s0001g08240.1
AT5G49660.1
CIL1055S0087
CIL1081S0043
CIL1055S0088
CIL0170S0013
AT3G19700.1
VIT 203s0088g01180.1
VIT 218s0001g08230.1
AT1G09970.2
AT4G28490.1
CIL1585S0002
VIT 212s0028g03060.1
Aco018064.1
Aco004473.1
VIT 210s0003g00330.1
AT1G28440.1
CIL0893S0368
CIL1086S0052
Aco005980.1
Aco009964.1
CIL0942S0152
CIL0282S0004
CIL1006S0055
VIT 211s0016g03080.1
VIT 204s0008g00330.1
VIT 204s0008g00300.1
VIT 204s0008g00310.1
VIT 204s0008g00440.1
VIT 204s0008g00920.1
VIT 204s0008g00410.1
VIT 204s0008g00430.1
VIT 204s0008g00420.1
VIT 204s0008g00390.1
VIT 204s0008g00370.1
VIT 204s0008g00350.1
VIT 204s0008g00400.1
VIT 204s0008g00290.1
VIT 215s0024g01270.1
CIL0987S0132
AT5G25930.1

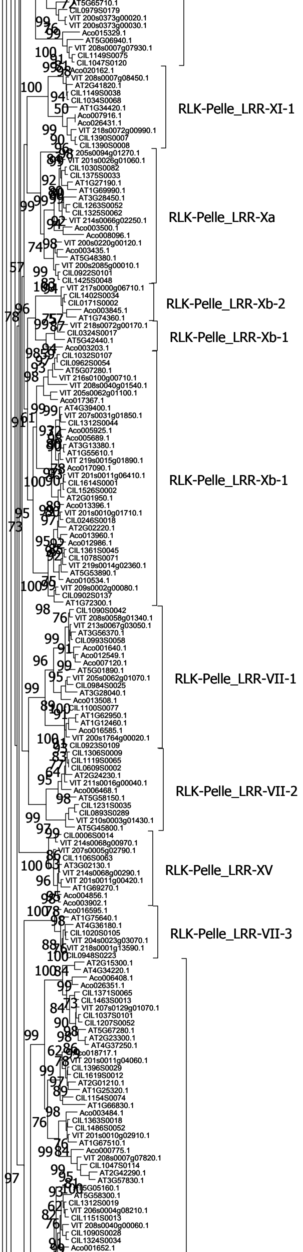
RLK-Pelle_LRR-XI-1
RLK-Pelle_LRR-Xa
RLK-Pelle_LRR-Xb-2
RLK-Pelle_LRR-Xb-1
RLK-Pelle_LRR-Xb-1
RLK-Pelle_LRR-VII-1
RLK-Pelle_LRR-VII-2
RLK-Pelle_LRR-XV
RLK-Pelle_LRR-VII-3

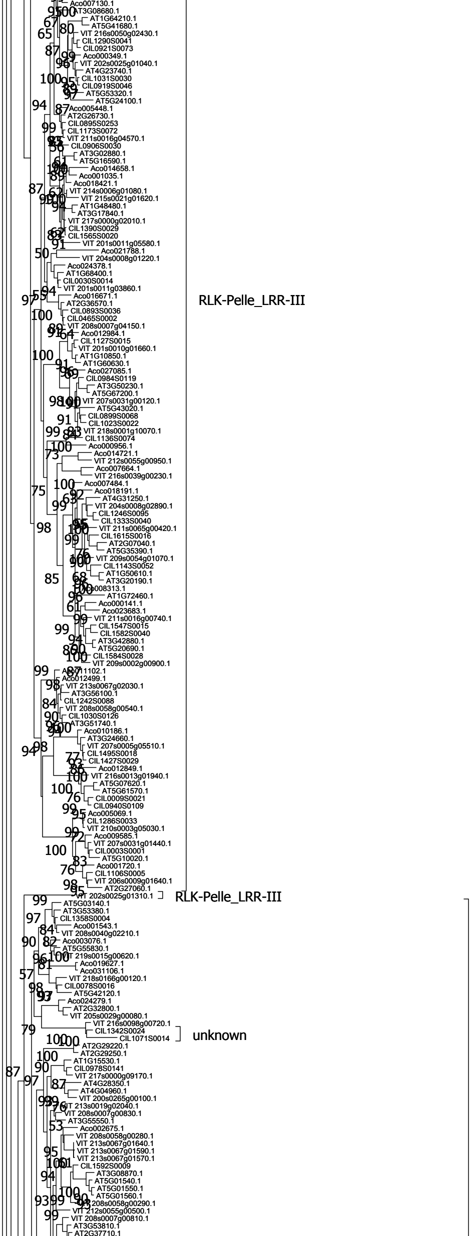

Aco007130.1
AT3G08680.1
AT1G64210.1
AT5G41680.1
VIT 216s0050g02430.1
CIL1290S0041
CIL0921S0073
Aco000349.1
VIT 202s0025g01040.1
AT4G23740.1
CIL1031S0030
CIL0919S0046
AT5G53320.1
AT5G24100.1
Aco005448.1
AT2G26730.1
CIL0895S0253
CIL1173S0072
VIT 211s0016g04570.1
CIL0906S0030
AT3G02880.1
AT5G16590.1
Aco014658.1
Aco001035.1
Aco018421.1
VIT 214s0006g01080.1
VIT 215s0021g01620.1
AT1G48480.1
AT3G17840.1
VIT 217s0000g02010.1
CIL1390S0029
CIL1565S0020
VIT 201s0011g05580.1
Aco021788.1
VIT 204s0008g01220.1
Aco024378.1
AT1G68400.1
CIL0030S0014
VIT 201s0011g03860.1
Aco016671.1
AT2G36570.1
CIL0893S0036
CIL0465S0002
VIT 208s0007g04150.1
Aco012984.1
CIL1127S0015
VIT 201s0010g01660.1
AT1G10850.1
AT1G60630.1
Aco027085.1
CIL0984S0119
AT3G50230.1
AT5G67200.1
VIT 207s0031g00120.1
AT5G43020.1
CIL0899S0068
CIL1023S0022
VIT 218s0001g10070.1
CIL1136S0074
Aco000956.1
Aco014721.1
VIT 212s0055g00950.1
Aco007664.1
VIT 216s0039g00230.1
Aco007484.1
Aco018191.1
AT4G31250.1
VIT 204s0008g02890.1
CIL1246S0095
CIL1333S0040
VIT 211s0065g00420.1
CIL1615S0016
AT2G07040.1
AT5G35390.1
VIT 209s0054g01070.1
CIL1143S0052
AT1G50610.1
AT3G20190.1
AT1G72460.1
Aco000141.1
Aco023683.1
VIT 211s0016g00740.1
CIL1547S0015
CIL1582S0040
AT3G42880.1
AT5G20690.1
CIL1584S0028
VIT 209s0002g00900.1
Aco012499.1
VIT 213s0067g02030.1
AT3G56100.1
CIL1242S0088
VIT 208s0058g00540.1
CIL1030S0126
AT3G51740.1
Aco010186.1
AT3G24660.1
VIT 207s0005g05510.1
CIL1495S0018
CIL1427S0029
Aco012849.1
VIT 216s0013g01940.1
AT5G07620.1
AT5G61570.1
CIL0009S0021
CIL0940S0109
Aco005069.1
CIL1286S0033
VIT 210s0003g05030.1
Aco009585.1
VIT 207s0031g01440.1
CIL0003S0001
AT5G10020.1
Aco001720.1
CIL1106S0005
VIT 206s0009g01640.1
AT2G27060.1
RLK-Pelle_LRR-III
VIT 202s0025g01310.1
RLK-Pelle_LRR-III
AT5G03140.1
AT3G53380.1
CIL1358S0004
Aco001543.1
VIT 208s0040g02210.1
Aco003076.1
AT5G55830.1
VIT 219s0015g00620.1
Aco019627.1
Aco031106.1
VIT 218s0166g00120.1
CIL0078S0016
AT5G42120.1
Aco024279.1
AT2G32800.1
VIT 205s0029g00080.1
VIT 216s0098g00720.1
CIL1342S0024
CIL1071S0014
unknown
AT2G29220.1
AT2G29250.1
AT1G15530.1
CIL0978S0141
VIT 217s0000g09170.1
AT4G28350.1
AT4G04960.1
VIT 200s0265g00100.1
VIT 213s0019g02040.1
VIT 208s0007g00830.1
AT3G55550.1
Aco002675.1
VIT 208s0058g00280.1
VIT 213s0067g01640.1
VIT 213s0067g01590.1
VIT 213s0067g01570.1
CIL1592S0009
AT3G08870.1
AT5G01540.1
AT5G01550.1
AT5G01560.1
VIT 208s0058g00290.1
VIT 212s0055g00500.1
VIT 208s0007g00810.1
AT3G53810.1
AT2G37710.1

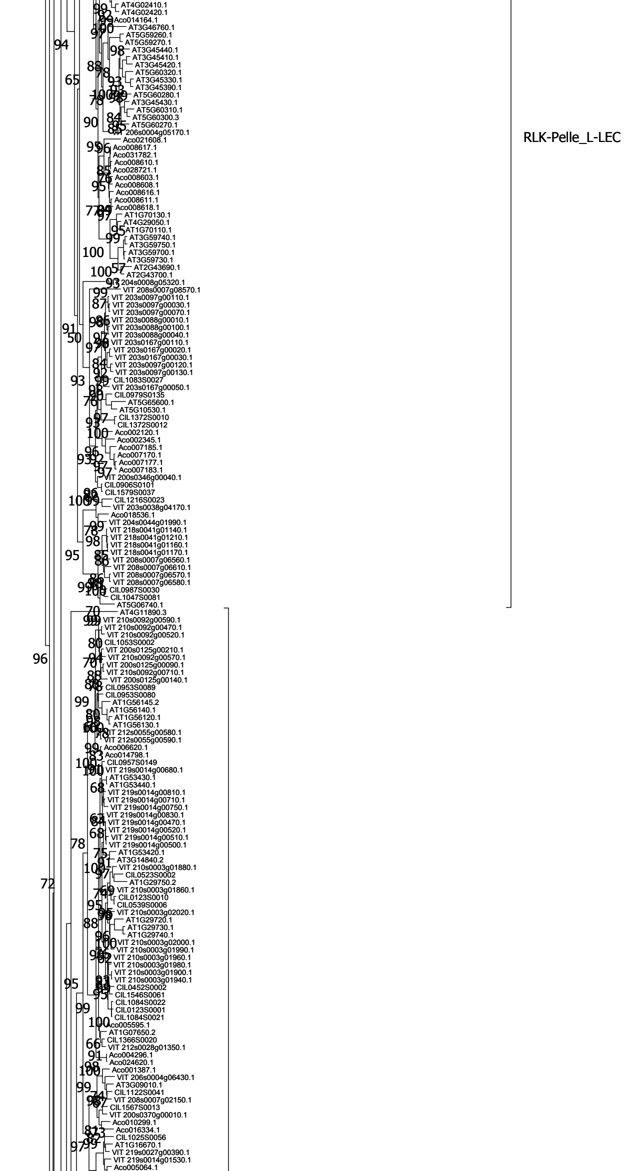
RLK-Pelle_L-LEC
AT4G02410.1
AT4G02420.1
Aco014164.1
AT3G46760.1
AT5G59260.1
AT5G59270.1
AT3G45440.1
AT3G45410.1
AT3G45420.1
AT5G60320.1
AT3G45330.1
AT3G45390.1
AT5G60280.1
AT3G45430.1
AT5G60310.1
AT5G60300.3
AT5G60270.1
VIT 206s0004g05170.1
Aco021608.1
Aco008617.1
Aco031782.1
Aco008610.1
Aco028721.1
Aco008603.1
Aco008608.1
Aco008616.1
Aco008611.1
Aco008618.1
AT1G70130.1
AT4G29050.1
AT1G70110.1
AT3G59740.1
AT3G59750.1
AT3G59700.1
AT3G59730.1
AT2G43690.1
AT2G43700.1
204s0008g05320.1
VIT 208s0007g08570.1
VIT 203s0097g00110.1
VIT 203s0097g00030.1
VIT 203s0097g00070.1
VIT 203s0088g00010.1
VIT 203s0088g00100.1
VIT 203s0088g00040.1
VIT 203s0167g00110.1
VIT 203s0167g00020.1
VIT 203s0167g00030.1
VIT 203s0097g00120.1
VIT 203s0097g00130.1
CIL1083S0027
VIT 203s0167g00050.1
CIL0979S0135
AT5G65600.1
AT5G10530.1
CIL1372S0010
CIL1372S0012
Aco002120.1
Aco002345.1
Aco007185.1
Aco007170.1
Aco007177.1
Aco007183.1
VIT 200s0346g00040.1
CIL0906S0101
CIL1579S0037
CIL1216S0023
VIT 203s0038g04170.1
Aco018536.1
VIT 204s0044g01990.1
VIT 218s0041g01140.1
VIT 218s0041g01210.1
VIT 218s0041g01160.1
VIT 218s0041g01170.1
VIT 208s0007g06560.1
VIT 208s0007g06610.1
VIT 208s0007g06570.1
VIT 208s0007g06580.1
CIL0987S0030
CIL1047S0081
AT5G06740.1
AT4G11890.3
VIT 210s0092g00590.1
VIT 210s0092g00470.1
VIT 210s0092g00520.1
CIL1053S0002
VIT 200s0125g00210.1
VIT 210s0092g00570.1
VIT 200s0125g00090.1
VIT 210s0092g00710.1
VIT 200s0125g00140.1
CIL0953S0089
CIL0953S0080
AT1G56145.2
AT1G56140.1
AT1G56120.1
AT1G56130.1
VIT 212s0055g00580.1
VIT 212s0055g00590.1
Aco006620.1
Aco014798.1
CIL0957S0149
VIT 219s0014g00680.1
AT1G53430.1
AT1G53440.1
VIT 219s0014g00810.1
VIT 219s0014g00710.1
VIT 219s0014g00750.1
VIT 219s0014g00830.1
VIT 219s0014g00470.1
VIT 219s0014g00520.1
VIT 219s0014g00510.1
VIT 219s0014g00500.1
AT1G53420.1
AT3G14840.2
VIT 210s0003g01880.1
CIL0523S0002
AT1G29750.2
VIT 210s0003g01860.1
CIL0123S0010
CIL0539S0006
VIT 210s0003g02020.1
AT1G29720.1
AT1G29730.1
AT1G29740.1
VIT 210s0003g02000.1
VIT 210s0003g01990.1
VIT 210s0003g01960.1
VIT 210s0003g01980.1
VIT 210s0003g01900.1
VIT 210s0003g01940.1
CIL0452S0002
CIL1546S0061
CIL1084S0022
CIL0123S0001
CIL1084S0021
Aco005595.1
AT1G07650.2
CIL1366S0020
VIT 212s0028g01350.1
Aco004296.1
Aco024620.1
Aco001387.1
VIT 206s0004g06430.1
AT3G09010.1
CIL1122S0041
VIT 208s0007g02150.1
CIL1567S0013
VIT 200s0370g00010.1
Aco010299.1
Aco016334.1
CIL1025S0056
AT1G16670.1
VIT 219s0027g00390.1
VIT 219s0014g01530.1
Aco005064.1

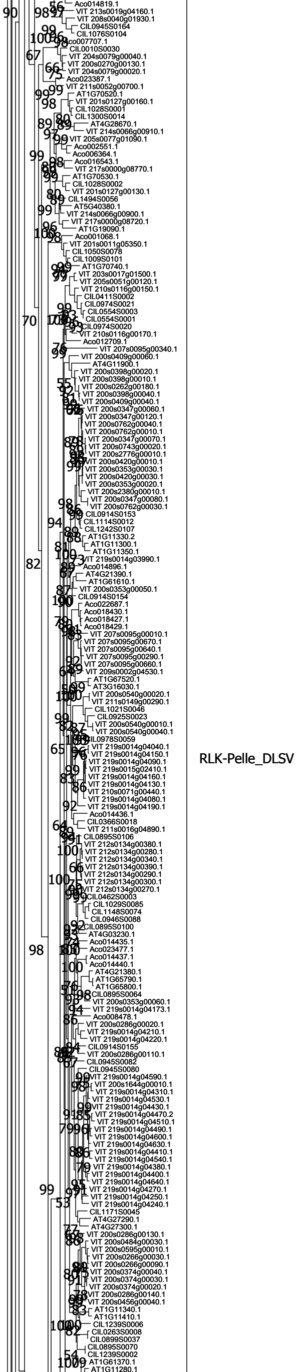
Aco014819.1
VIT 213s0019g04160.1
VIT 208s0040g01930.1
CIL0945S0164
CIL1076S0104
Aco007707.1
CIL0010S0030
VIT 204s0079g00040.1
VIT 200s0270g00130.1
VIT 204s0079g00020.1
Aco023387.1
VIT 211s0052g00700.1
AT1G70520.1
VIT 201s0127g00160.1
CIL1028S0001
CIL1300S0014
AT4G28670.1
VIT 214s0066g00910.1
VIT 205s0077g01090.1
Aco002551.1
Aco006364.1
Aco016543.1
VIT 217s0000g08770.1
AT1G70530.1
CIL1028S0002
VIT 201s0127g00130.1
CIL1494S0056
AT5G40380.1
VIT 214s0066g00900.1
VIT 217s0000g08720.1
AT1G19090.1
Aco001068.1
VIT 201s0011g05350.1
CIL1050S0078
CIL1009S0101
AT1G70740.1
VIT 203s0017g01500.1
VIT 205s0051g00120.1
VIT 210s0116g00150.1
CIL0411S0002
CIL0974S0021
CIL0554S0003
CIL0554S0001
CIL0974S0020
VIT 210s0116g00170.1
Aco012709.1
VIT 207s0095g00340.1
VIT 200s0409g00060.1
AT4G11900.1
VIT 200s0398g00020.1
VIT 200s0398g00010.1
VIT 200s0262g00180.1
VIT 200s0398g00040.1
VIT 200s0409g00040.1
VIT 200s0347g00060.1
VIT 200s0347g00120.1
VIT 200s0762g00040.1
VIT 200s0762g00010.1
VIT 200s0347g00070.1
VIT 200s0743g00020.1
VIT 200s2776g00010.1
VIT 200s0420g00010.1
VIT 200s0353g00030.1
VIT 200s0420g00030.1
VIT 200s0353g00020.1
VIT 200s2380g00010.1
VIT 200s0347g00080.1
VIT 200s0762g00030.1
CIL0914S0153
CIL1114S0012
CIL1242S0107
AT1G11330.2
AT1G11300.1
AT1G11350.1
VIT 219s0014g03990.1
Aco014896.1
AT4G21390.1
AT1G61610.1
VIT 200s0353g00050.1
CIL0914S0154
Aco022687.1
Aco018430.1
Aco018427.1
Aco018429.1
VIT 207s0095g00010.1
VIT 207s0095g00670.1
VIT 207s0095g00640.1
VIT 207s0095g00290.1
VIT 207s0095g00660.1
VIT 209s0002g04530.1
AT1G67520.1
AT3G16030.1
VIT 200s0540g00020.1
VIT 211s0149g00290.1
CIL1021S0046
CIL0925S0023
VIT 200s0540g00010.1
VIT 200s0540g00040.1
CIL0978S0059
VIT 219s0014g04040.1
VIT 219s0014g04150.1
VIT 219s0014g04090.1
VIT 219s0015g02410.1
VIT 219s0014g04160.1
VIT 219s0014g04130.1
VIT 210s0071g00440.1
VIT 219s0014g04080.1
VIT 219s0014g04190.1
Aco014436.1
CIL0366S0018
VIT 211s0016g04890.1
CIL0895S0106
VIT 212s0134g00380.1
VIT 212s0134g00280.1
VIT 212s0134g00340.1
VIT 212s0134g00390.1
VIT 212s0134g00290.1
VIT 212s0134g00300.1
VIT 212s0134g00270.1
CIL0462S0003
CIL1029S0085
CIL1148S0074
CIL0946S0088
CIL0895S0100
AT4G03230.1
Aco014435.1
Aco023477.1
Aco014437.1
Aco014440.1
AT4G21380.1
AT1G65790.1
AT1G65800.1
CIL0895S0064
VIT 200s0353g00060.1
VIT 219s0014g04173.1
Aco008478.1
VIT 200s0286g00020.1
VIT 219s0014g04210.1
VIT 219s0014g04220.1
CIL0914S0155
VIT 200s0286g00110.1
CIL0945S0082
CIL0945S0080
VIT 219s0014g04590.1
VIT 200s1644g00010.1
VIT 219s0014g04310.1
VIT 219s0014g04530.1
VIT 219s0014g04430.1
VIT 219s0014g04470.2
VIT 219s0014g04510.1
VIT 219s0014g04490.1
VIT 219s0014g04600.1
VIT 219s0014g04630.1
VIT 219s0014g04410.1
VIT 219s0014g04540.1
VIT 219s0014g04380.1
VIT 219s0014g04400.1
VIT 219s0014g04640.1
VIT 219s0014g04270.1
VIT 219s0014g04250.1
VIT 219s0014g04240.1
CIL1171S0045
AT4G27290.1
AT4G27300.1
VIT 200s0286g00130.1
VIT 200s0484g00030.1
VIT 200s0595g00010.1
VIT 200s0266g00030.1
VIT 200s0266g00090.1
VIT 200s0374g00040.1
VIT 200s0374g00030.1
VIT 200s0374g00020.1
VIT 200s0286g00140.1
VIT 200s0456g00040.1
AT1G11340.1
AT1G11410.1
CIL1239S0006
CIL0263S0008
CIL0899S0037
CIL0895S0070
CIL1239S0002
AT1G61370.1
AT1G11280.1
RLK-Pelle_DLSV

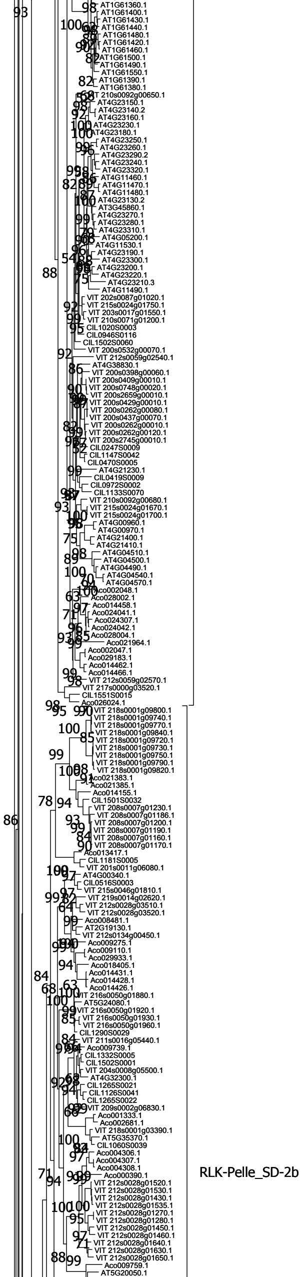
AT1G61360.1
AT1G61400.1
AT1G61430.1
AT1G61440.1
AT1G61480.1
AT1G61420.1
AT1G61460.1
AT1G61500.1
AT1G61490.1
AT1G61550.1
AT1G61390.1
AT1G61380.1
VIT 210s0092g00650.1
AT4G23150.1
AT4G23140.2
AT4G23160.1
AT4G23230.1
AT4G23180.1
AT4G23250.1
AT4G23260.1
AT4G23290.2
AT4G23240.1
AT4G23320.1
AT4G11460.1
AT4G11470.1
AT4G11480.1
AT4G23130.2
AT3G45860.1
AT4G23270.1
AT4G23280.1
AT4G23310.1
AT4G05200.1
AT4G11530.1
AT4G23190.1
AT4G23300.1
AT4G23200.1
AT4G23220.1
AT4G23210.3
AT4G11490.1
VIT 202s0087g01020.1
VIT 215s0024g01750.1
VIT 203s0017g01550.1
VIT 210s0071g01200.1
CIL1020S0003
CIL0946S0116
CIL1502S0060
VIT 200s0532g00070.1
VIT 212s0059g02540.1
AT4G38830.1
VIT 200s0398g00060.1
VIT 200s0409g00010.1
VIT 200s0748g00020.1
VIT 200s2659g00010.1
VIT 200s0429g00010.1
VIT 200s0262g00080.1
VIT 200s0437g00070.1
VIT 200s0262g00010.1
VIT 200s0262g00120.1
VIT 200s2745g00010.1
CIL0247S0009
CIL1147S0042
CIL0470S0005
AT4G21230.1
CIL0419S0009
CIL0972S0002
CIL1133S0070
VIT 210s0092g00680.1
VIT 215s0024g01670.1
VIT 215s0024g01700.1
AT4G00960.1
AT4G00970.1
AT4G21400.1
AT4G21410.1
AT4G04510.1
AT4G04500.1
AT4G04490.1
AT4G04540.1
AT4G04570.1
Aco002048.1
Aco028002.1
Aco014458.1
Aco024041.1
Aco024307.1
Aco024042.1
Aco028004.1
Aco021964.1
Aco002047.1
Aco029183.1
Aco014462.1
Aco014466.1
VIT 212s0059g02570.1
VIT 217s0000g03520.1
CIL1551S0015
Aco026024.1
VIT 218s0001g09800.1
VIT 218s0001g09740.1
VIT 218s0001g09770.1
VIT 218s0001g09840.1
VIT 218s0001g09720.1
VIT 218s0001g09730.1
VIT 218s0001g09750.1
VIT 218s0001g09790.1
VIT 218s0001g09820.1
Aco021383.1
Aco021385.1
Aco014155.1
CIL1501S0032
VIT 208s0007g01230.1
VIT 208s0007g01186.1
VIT 208s0007g01200.1
VIT 208s0007g01190.1
VIT 208s0007g01160.1
VIT 208s0007g01170.1
Aco013417.1
CIL1181S0005
VIT 201s0011g06080.1
AT4G00340.1
CIL0516S0003
VIT 215s0046g01810.1
VIT 219s0014g02620.1
VIT 212s0028g03510.1
VIT 212s0028g03520.1
Aco008481.1
AT2G19130.1
VIT 212s0134g00450.1
Aco009275.1
Aco009110.1
Aco029933.1
Aco018405.1
Aco014431.1
Aco014428.1
Aco014426.1
VIT 216s0050g01880.1
AT5G24080.1
VIT 216s0050g01920.1
VIT 216s0050g01930.1
VIT 216s0050g01960.1
CIL1290S0029
VIT 211s0016g05440.1
Aco009739.1
CIL1332S0005
CIL1502S0001
VIT 204s0008g05500.1
AT4G32300.1
CIL1265S0021
CIL1126S0041
CIL1265S0022
VIT 209s0002g06830.1
Aco001333.1
Aco002681.1
VIT 218s0001g03390.1
AT5G35370.1
CIL1060S0039
Aco004306.1
Aco004307.1
Aco004308.1
Aco000390.1
RLK-Pelle_SD-2b
VIT 212s0028g01520.1
VIT 212s0028g01530.1
VIT 212s0028g01430.1
VIT 212s0028g01535.1
VIT 212s0028g01270.1
VIT 212s0028g01280.1
VIT 212s0028g01450.1
VIT 212s0028g01460.1
VIT 212s0028g01640.1
VIT 212s0028g01630.1
VIT 212s0028g01650.1
Aco009759.1
AT5G20050.1

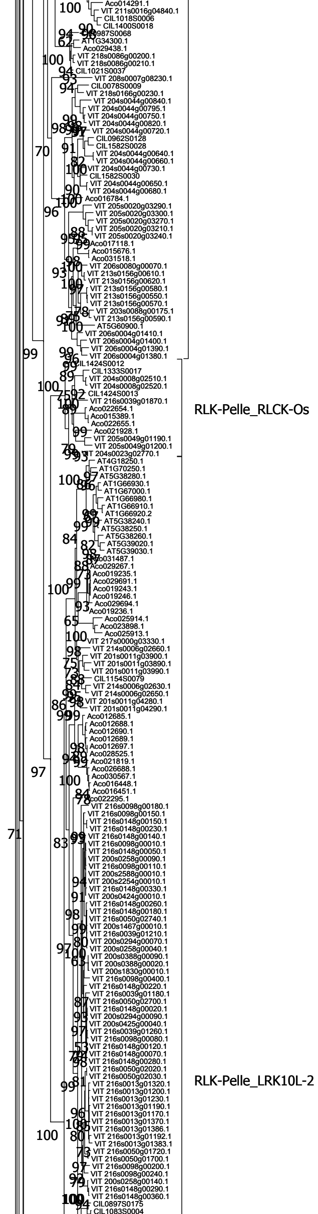
RLK-Pelle_RLCK-Os
RLK-Pelle_LRK10L-2

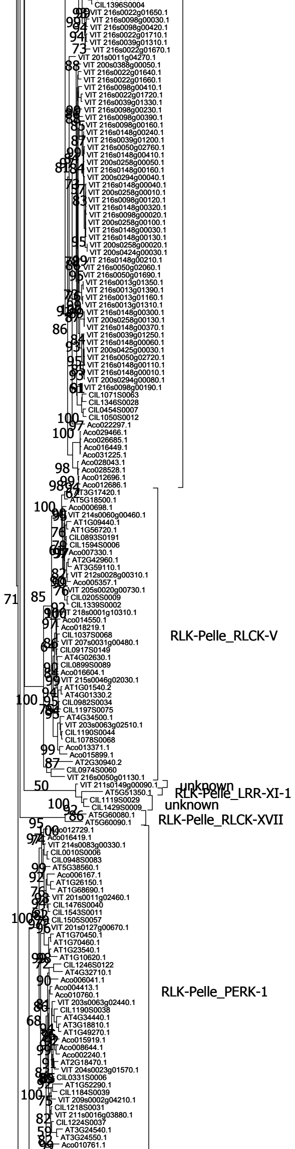
CIL1396S0004
VIT 216s0022g01650.1
VIT 216s0098g00030.1
VIT 216s0098g00420.1
VIT 216s0022g01710.1
VIT 216s0039g01310.1
VIT 216s0022g01670.1
VIT 201s0011g04270.1
VIT 200s0388g00050.1
VIT 216s0022g01640.1
VIT 216s0022g01660.1
VIT 216s0098g00410.1
VIT 216s0022g01720.1
VIT 216s0039g01330.1
VIT 216s0098g00230.1
VIT 216s0098g00390.1
VIT 216s0098g00160.1
VIT 216s0148g00240.1
VIT 216s0039g01200.1
VIT 216s0050g02760.1
VIT 216s0148g00410.1
VIT 200s0258g00050.1
VIT 216s0148g00160.1
VIT 200s0294g00040.1
VIT 216s0148g00040.1
VIT 200s0258g00010.1
VIT 216s0098g00120.1
VIT 216s0148g00320.1
VIT 216s0098g00020.1
VIT 200s0258g00100.1
VIT 216s0148g00030.1
VIT 216s0148g00130.1
VIT 200s0258g00020.1
VIT 200s0424g00030.1
VIT 216s0148g00210.1
VIT 216s0050g02060.1
VIT 216s0050g01690.1
VIT 216s0013g01350.1
VIT 216s0013g01390.1
VIT 216s0013g01160.1
VIT 216s0013g01310.1
VIT 216s0148g00300.1
VIT 200s0258g00130.1
VIT 216s0148g00370.1
VIT 216s0039g01250.1
VIT 216s0148g00060.1
VIT 200s0425g00030.1
VIT 216s0050g02720.1
VIT 216s0148g00110.1
VIT 216s0148g00010.1
VIT 200s0294g00080.1
VIT 216s0098g00190.1
CIL1071S0063
CIL1346S0028
CIL0454S0007
CIL1050S0012
Aco022297.1
Aco029466.1
Aco026685.1
Aco016449.1
Aco031225.1
Aco028043.1
Aco028528.1
Aco012696.1
Aco012686.1
AT3G17420.1
AT5G18500.1
Aco000698.1
VIT 214s0060g00460.1
AT1G09440.1
AT1G56720.1
CIL0893S0191
CIL1594S0006
Aco007330.1
AT2G42960.1
AT3G59110.1
VIT 212s0028g00310.1
Aco005357.1
VIT 205s0020g00730.1
CIL0205S0009
CIL1339S0002
VIT 218s0001g10310.1
Aco014550.1
Aco018219.1
CIL1037S0068
VIT 207s0031g00480.1
CIL0917S0149
AT4G02630.1
CIL0899S0089
Aco016604.1
VIT 215s0046g02030.1
AT1G01540.2
AT4G01330.2
CIL0982S0034
CIL1197S0075
AT4G34500.1
VIT 203s0063g02510.1
CIL1190S0044
CIL1078S0068
Aco013371.1
Aco015899.1
AT2G30940.2
CIL0974S0060
VIT 216s0050g01130.1
RLK-Pelle_RLCK-V
VIT 211s0149g00090.1
unknown
AT5G51350.1
RLK-Pelle_LRR-XI-1
CIL1119S0029
CIL1429S0009
unknown
AT5G60080.1
AT5G60090.1
RLK-Pelle_RLCK-XVII
Aco012729.1
Aco016419.1
VIT 214s0083g00330.1
CIL0010S0006
CIL0948S0083
AT5G38560.1
Aco006167.1
AT1G26150.1
AT1G68690.1
VIT 201s0011g02460.1
CIL1476S0040
CIL1543S0011
CIL1505S0057
VIT 201s0127g00670.1
AT1G70450.1
AT1G70460.1
AT1G23540.1
AT1G10620.1
CIL1246S0122
AT4G32710.1
Aco006041.1
Aco004413.1
Aco010760.1
VIT 203s0063g02440.1
CIL1190S0038
AT4G34440.1
AT3G18810.1
AT1G49270.1
Aco015919.1
Aco008644.1
Aco002240.1
AT2G18470.1
VIT 204s0023g01570.1
CIL0331S0006
AT1G52290.1
CIL1184S0039
VIT 209s0002g04210.1
CIL1218S0031
VIT 211s0016g03880.1
CIL1224S0037
AT3G24540.1
AT3G24550.1
Aco010761.1
RLK-Pelle_PERK-1

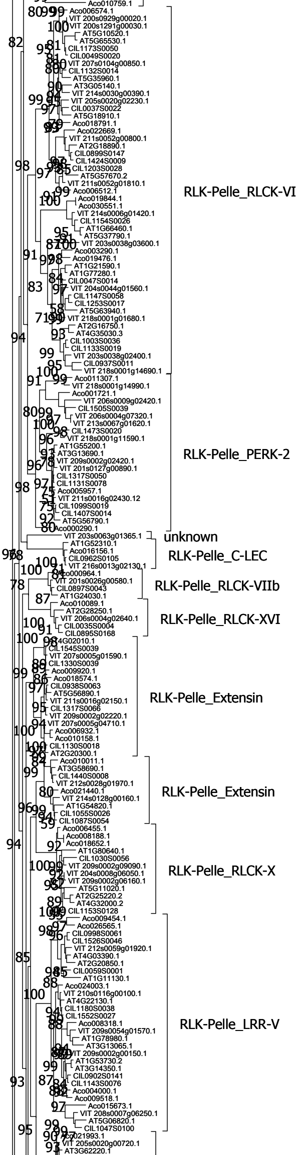
Aco010759.1
Aco006574.1
VIT 200s0929g00020.1
VIT 200s1291g00030.1
AT5G10520.1
AT5G65530.1
CIL1173S0050
CIL0049S0020
VIT 207s0104g00850.1
CIL1132S0014
AT5G35960.1
AT3G05140.1
VIT 214s0030g00390.1
VIT 205s0020g02230.1
CIL0037S0022
AT5G18910.1
Aco018791.1
Aco022669.1
VIT 211s0052g00800.1
AT2G18890.1
CIL0899S0147
CIL1424S0009
CIL1203S0028
AT5G57670.2
VIT 211s0052g01810.1
Aco006512.1
Aco019844.1
Aco030551.1
VIT 214s0006g01420.1
CIL1154S0026
AT1G66460.1
AT5G37790.1
VIT 203s0038g03600.1
Aco003290.1
Aco019476.1
AT1G21590.1
AT1G77280.1
CIL0047S0014
VIT 204s0044g01560.1
CIL1147S0058
CIL1253S0017
AT5G63940.1
VIT 218s0001g01680.1
AT2G16750.1
AT4G35030.3
CIL1003S0036
CIL1133S0019
VIT 203s0038g02400.1
CIL0937S0011
VIT 218s0001g14690.1
Aco011307.1
VIT 218s0001g14990.1
Aco001721.1
VIT 206s0009g02420.1
CIL1505S0039
VIT 206s0004g07320.1
VIT 213s0067g01620.1
CIL1473S0020
VIT 218s0001g11590.1
AT1G55200.1
AT3G13690.1
VIT 209s0002g02420.1
VIT 201s0127g00890.1
CIL1317S0050
CIL1131S0078
Aco005957.1
VIT 211s0016g02430.12
CIL1099S0019
CIL1407S0014
AT5G56790.1
Aco000290.1
VIT 203s0063g01365.1
AT1G52310.1
Aco016156.1
CIL0962S0105
VIT 216s0013g02130.1
Aco000964.1
VIT 201s0026g00580.1
CIL0897S0043
AT1G24030.1
Aco010089.1
AT2G28250.1
VIT 206s0004g02640.1
CIL0035S0004
CIL0895S0168
AT4G02010.1
CIL1545S0039
VIT 207s0005g01590.1
CIL1330S0039
Aco009920.1
Aco018574.1
CIL0938S0063
AT5G56890.1
VIT 211s0016g02150.1
CIL1317S0066
VIT 209s0002g02220.1
VIT 207s0005g04710.1
Aco006932.1
Aco010158.1
CIL1130S0018
AT2G20300.1
Aco010011.1
AT3G58690.1
CIL1440S0008
VIT 212s0028g01970.1
Aco021440.1
VIT 214s0128g00160.1
AT1G54820.1
CIL1055S0026
CIL1087S0054
Aco006455.1
Aco008188.1
Aco018652.1
AT1G80640.1
CIL1030S0056
VIT 209s0002g09090.1
VIT 204s0008g06050.1
VIT 209s0002g06160.1
AT5G11020.1
AT2G25220.2
AT4G32000.2
CIL1153S0128
Aco009454.1
Aco026565.1
CIL0998S0061
CIL1526S0046
VIT 212s0059g01920.1
AT4G03390.1
AT2G20850.1
CIL0059S0001
AT1G11130.1
Aco024003.1
VIT 210s0116g00100.1
AT4G22130.1
CIL1180S0038
CIL1552S0027
Aco008318.1
VIT 209s0054g01570.1
AT1G78980.1
AT3G13065.1
VIT 209s0002g00150.1
AT1G53730.2
AT3G14350.1
CIL0902S0141
CIL1143S0076
Aco004000.1
Aco009518.1
Aco015673.1
VIT 208s0007g06250.1
AT5G06820.1
CIL1047S0100
Aco021993.1
VIT 205s0020g00720.1
AT3G62220.1
RLK-Pelle_RLCK-VI
RLK-Pelle_PERK-2
unknown
RLK-Pelle_C-LEC
RLK-Pelle_RLCK-VIIb
RLK-Pelle_RLCK-XVI
RLK-Pelle_Extensin
RLK-Pelle_Extensin
RLK-Pelle_RLCK-X
RLK-Pelle_LRR-V

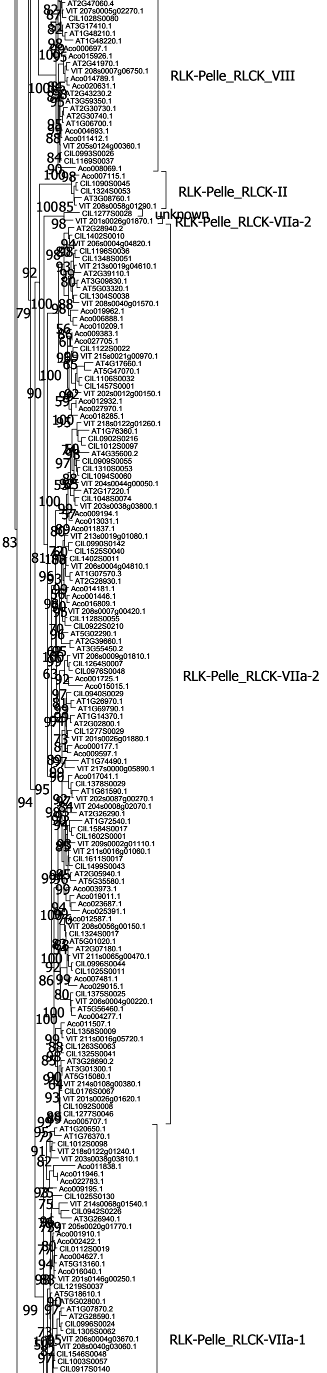
RLK-Pelle_RLCK_VIII
RLK-Pelle_RLCK-II
unknown
RLK-Pelle_RLCK-VIIa-2
RLK-Pelle_RLCK-VIIa-2
RLK-Pelle_RLCK-VIIa-1

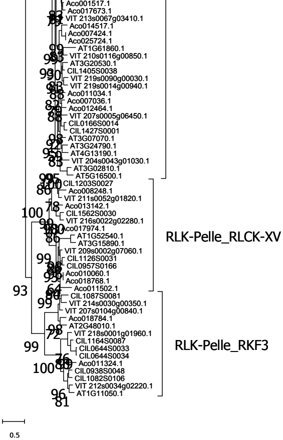

Aco001517.1
Aco017673.1
VIT 213s0067g03410.1
Aco014517.1
Aco007424.1
Aco025724.1
AT1G61860.1
VIT 210s0116g00850.1
AT3G20530.1
CIL1405S0038
VIT 219s0090g00030.1
VIT 219s0014g00940.1
Aco011034.1
Aco007036.1
Aco012464.1
VIT 207s0005g06450.1
CIL0166S0014
CIL1427S0001
AT3G07070.1
AT3G24790.1
AT4G13190.1
VIT 204s0043g01030.1
AT3G02810.1
AT5G16500.1
CIL1203S0027
Aco008248.1
VIT 211s0052g01820.1
Aco013142.1
CIL1562S0030
VIT 216s0022g02280.1
Aco017974.1
AT1G52540.1
AT3G15890.1
VIT 209s0002g07060.1
CIL1126S0031
CIL0957S0166
Aco010060.1
Aco018768.1
Aco011502.1
CIL1087S0081
VIT 214s0030g00350.1
VIT 207s0104g00840.1
Aco018784.1
AT2G48010.1
VIT 218s0001g01960.1
CIL1164S0087
CIL0644S0033
CIL0644S0034
Aco011324.1
CIL0938S0048
CIL1082S0106
VIT 212s0034g02220.1
AT1G11050.1
RLK-Pelle_RLCK-XV
RLK-Pelle_RKF3
0.5
